# Noncanonical Rab9a action supports retromer-mediated endosomal exit of human papillomavirus during virus entry

**DOI:** 10.1371/journal.ppat.1011648

**Published:** 2023-09-13

**Authors:** Jeongjoon Choi, Daniel DiMaio

**Affiliations:** 1 Department of Genetics, Yale School of Medicine, New Haven, Connecticut, United States of America; 2 Department of Therapeutic Radiology, Yale School of Medicine, New Haven, Connecticut, United States of America; 3 Department of Molecular Biophysics & Biochemistry, Yale University, New Haven, Connecticut, United States of America; 4 Yale Cancer Center, New Haven, Connecticut, United States of America; Penn State University School of Medicine, UNITED STATES

## Abstract

Rab GTPases play key roles in controlling intracellular vesicular transport. GTP-bound Rab proteins support vesicle trafficking. Here, we report that, unlike cellular protein cargos, retromer-mediated delivery of human papillomaviruses (HPV) into the retrograde transport pathway during virus entry is inhibited by Rab9a in its GTP-bound form. Knockdown of Rab9a inhibits HPV entry by modulating the HPV-retromer interaction and impairing retromer-mediated endosome-to-Golgi transport of the incoming virus, resulting in the accumulation of HPV in the endosome. Rab9a is in proximity to HPV as early as 3.5 h post-infection, prior to the Rab7-HPV interaction, and HPV displays increased association with retromer in Rab9a knockdown cells, even in the presence of dominant negative Rab7. Thus, Rab9a can regulate HPV-retromer association independently of Rab7. Surprisingly, excess GTP-Rab9a impairs HPV entry, whereas excess GDP-Rab9a reduces association between L2 and Rab9a and stimulates entry. These findings reveal that HPV and cellular proteins utilize the Rab9a host trafficking machinery in distinct ways during intracellular trafficking.

## Author summary

Rab GTPases are small proteins that regulate transport of membrane-bound vesicles inside cells. Canonically, guanosine triphosphate (GTP)-bound Rab proteins support vesicle trafficking. Here, we report that, unlike cellular protein cargos, GTP-bound Rab9a inhibits the entry of human papillomaviruses (HPV) as the virus travels inside vesicles to the nucleus. HPV are small non-enveloped DNA viruses that trigger ~5% of human cancer. During HPV entry, Rab9a associates with the L2 protein in the virus particle. Depletion of Rab9a inhibits HPV infection by modulating the interaction of HPV with retromer, a cellular protein trafficking complex, and impairing retromer-mediated endosome-to-Golgi trafficking of the incoming virus during virus entry. This HPV trafficking step also requires Rab7, which acts later than Rab9a. Surprisingly, excess GTP-bound Rab9a impairs HPV entry, whereas excess guanosine diphosphate (GDP)-bound Rab9a stimulates the entry. The HPV L2 protein initially associates with GTP-Rab9a, but when Rab9a is converted to the GDP-bound form, L2 is transferred to Rab7 for further trafficking. Our findings reveal that HPV and cellular proteins utilize the Rab9a host trafficking machinery in distinct ways during intracellular trafficking, providing a potential vulnerability for therapeutic approaches.

## Introduction

Rab GTPases are key regulators of intracellular vesicular transport [1–3]. In general, when bound to guanosine triphosphate (GTP), Rab proteins are active and support vesicle trafficking, but when bound to guanosine diphosphate (GDP) they do not support vesicle transport [1,3]. Trafficking of some viruses to the site of viral genome replication depends on cellular Rab proteins [4–6]. Human papillomaviruses (HPVs) are non-enveloped, double-stranded DNA viruses that trigger ~5% of human cancer, including essentially all cervical cancer [7]. HPV entry requires various Rab proteins, but it remains largely unclear which Rab proteins are employed at which specific steps of virus entry [6]. Here, we show that GDP-bound Rab9a is necessary for HPV trafficking from the endosome to the trans-Golgi network (TGN) during entry.

The HPV virion contains 360 molecules of the major capsid protein, L1, and up to 72 molecules of the minor capsid protein, L2 [8]. L1 is responsible for HPV binding to the cell surface, while L2 is essential for trafficking of the viral genome to the nucleus where viral gene expression and DNA replication occur [9]. Upon virus internalization, viral components including viral DNA reside within vesicular retrograde trafficking compartments throughout the entry process [10,11]. After initially localizing in endosomes, HPV traffics to the *trans*-Golgi network (TGN) in a manner dependent on retromer, a cellular protein sorting complex [12,13]. HPV then traffics through the Golgi apparatus and possibly the endoplasmic reticulum *en route* to the nucleus [9,12–15]. During entry, a portion of the L2 protein protrudes through the endosome membrane into the cytoplasm, allowing it to interact with various cellular proteins to enable retrograde trafficking of the incoming virus particle [16,17]. Cytoplasmic proteins involved in HPV retrograde trafficking that interact with L2 include retromer [13], sorting nexin 17 and 27 [18,19], dynein [20], retriever [21], and COPI [22].

Retromer, a three-subunit protein complex (VPS26, VPS29, and VPS35), binds directly to the C-terminus of L2 and plays a critical role in endosome-to-TGN transport of HPV [12,13]. GTP-Rab7 interacts with retromer [23] and recruits and stabilizes the association of HPV with retromer at the endosomal membrane [24]. In addition, cycling between GTP- and GDP-bound Rab7 is critical for endosome-to-TGN trafficking of HPV: while GTP-Rab7 recruits retromer, GTP hydrolysis to generate GDP-Rab7 is required for dissociation of the retromer from HPV, which must occur for trafficking to proceed [24]. Rab9a is also required for HPV entry, but its role in this process has not been studied [12,15]. Because Rab9a facilitates endosome-to-TGN trafficking of cellular proteins [25], it is plausible that it acts at a similar step in HPV entry.

In this study, we show that Rab9a supports HPV type 16 (HPV16) entry by associating with incoming HPV early during infection and modulating the association of retromer and HPV. This activity of Rab9a is critical for endosome exit of HPV. Rab9a engages HPV prior to Rab7 and modulates the HPV-retromer association even in cells expressing dominant negative Rab7. Surprisingly, unlike cellular protein cargos, Rab9a-mediated HPV trafficking is promoted by an increased GDP-Rab9a to GTP-Rab9a ratio and inhibited by an increased ratio of GTP-Rab9a to GDP-Rab9a. Our findings reveal a vesicle transport mechanism facilitated by GDP-bound Rab, distinct from cellular protein cargo trafficking.

## Results

### Rab9a is required for virus trafficking from endosomes to Golgi during HPV entry

**Fig 1:**
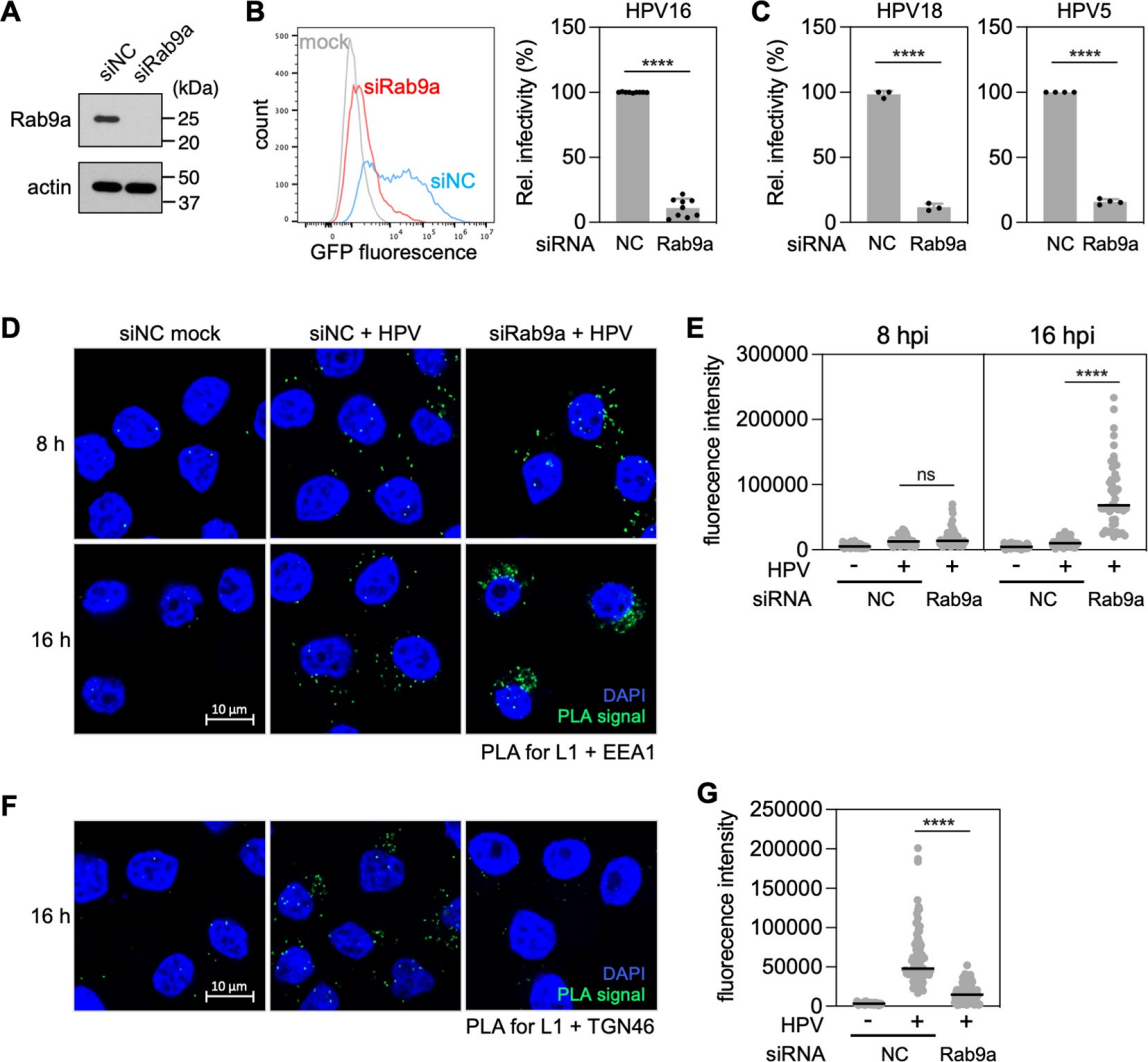
Rab9a knockdown inhibits HPV infection by impairing its trafficking from endosomes to Golgi. (**A**) HeLa S3 cells were transfected with negative control (siNC) or Rab9a-targeting siRNA (siRab9a) and subjected to Western blot analysis using an antibody recognizing Rab9a (top panel) and actin (bottom panel) as a loading control. (**B**) siRNA-treated cells as described in (**A**) were mock-infected or infected at the MOI of ~2 with HPV16 PsV L2-3XFLAG containing the GFP reporter plasmid. At 48 hpi, GFP fluorescence was determined by flow cytometry. The results are shown as a histogram (*left*) and as percent relative infectivity (based on mean fluorescence intensity) normalized to the siNC treated cells (*right*). Each dot shows the result of an individual experiment. Bars and error bars show mean and standard deviation, respectively. NC, siNC. ****, *P* < 0.0001. (**C**) As in (**B**) except cells were infected with HPV18 and HPV5. (**D**) HeLa S3 cells were transfected with siNC or siRab9a siRNAs and infected with HPV harboring the HcRed reporter plasmid at the MOI of ~200. At 8 and 16 hpi, PLA was performed with antibodies recognizing HPV L1 and EEA1. Mock, uninfected; HPV, infected. PLA signals are green; nuclei are blue (DAPI). Similar results were obtained in two independent experiments. (**E**) The fluorescence of PLA signals was determined from multiple images obtained as in (**D**). Each dot represents an individual cell (*n*>40) and black horizontal lines indicate the mean value of the analyzed population in each group. ****, *P* < 0.0001; ns, not significant. The graph shows results of a representative experiment. Similar results were obtained in two independent experiments. (**F**) As in (**D**) except PLA was performed at 16 hpi with antibodies recognizing HPV L1 and TGN46. (**G**) Images as in (**F**) were analyzed as described in (**E**).

Although Rab9a is required for efficient infection by HPV pseudovirus (PsV) [12,15], its role in this process has not been investigated. To determine the role of Rab9a in HPV infection, HeLa S3 cervical cancer cells were transfected with non-targeting control siRNA (siNC) and two different siRNAs targeting Rab9a expression (siRab9a and siRNA9a-2). Rab9a knockdown was confirmed by Western blotting (Figs 1A and S1A). These cells were then infected with HPV16 PsV consisting of a complete L1 and L2 capsid containing a reporter plasmid expressing GFP. A 3xFLAG tag was appended to the C-terminus of L2. Infectivity was determined by flow cytometry for GFP fluorescence at 48 h post infection (hpi). This assay measures only the entry phase of the HPV life cycle (which we define as virus internalization and trafficking to the nucleus). Consistent with our previously published results [12], knockdown of Rab9a by either Rab9a siRNA inhibited HPV infectivity (by >80% in the case of siRab9a) (Figs 1B and S1B). Infectivity of HPV18 and HPV5 PsV was reduced to a similar extent in HeLa cells depleted for Rab9a (Fig 1C). Rab9a knockdown also inhibited HPV16 PsV infection by ~80% in human HaCaT skin keratinocytes (S1C and S1D Fig). These data show that Rab9a is necessary for efficient entry of several pathogenic HPV types.

To determine the HPV entry step impaired by Rab9a knockdown, we used proximity ligation assay (PLA) to examine HPV localization in cells infected with HPV16 PsV. PLA generates a fluorescent signal in intact cells when two proteins of interest are within 40 nm [26]. Because Rab9a is necessary for cellular protein trafficking from the endosome to Golgi [25], we examined the proximity of HPV L1 and marker proteins for the endosome (EEA1) or TGN (TGN46). There were negligible PLA signals in mock infected cells. At 8 hpi, similar levels of L1-EEA1 PLA signals were observed in infected cells transfected with either siNC or siRab9a (Fig 1D and 1E), showing that Rab9a is not required for HPV endocytosis. In contrast, at 16 hpi, a time when most HPV has largely exited the endosome and entered the TGN in normal cells [13,14], Rab9a knockdown resulted in much stronger L1-EEA1 PLA signals than in the control cells, indicating that HPV accumulates in endosomes in the absence of Rab9a (Fig 1D and 1E). Consistent with this result, at 16 hpi control infected cells displayed strong L1-TGN46 PLA signals, as expected, whereas Rab9a knockdown cells showed little signal (Fig 1F and 1G), indicating that HPV arrival in TGN was impaired in the absence of Rab9a. Endosome accumulation and reduced HPV TGN localization in the Rab9a knockdown cells were also observed when antibody recognizing L2 rather than L1 was used for PLA (S2A-S2D Fig). We also tested if Rab9a knockdown resulted in increased HPV delivery to the lysosome. PLA signals for L2-LAMP1 (a marker protein for lysosome) interaction at 24 hpi were similar regardless of Rab9a knockdown (S2E and S2F Fig). Collectively, these data indicate that Rab9a supports HPV entry by enabling trafficking of HPV from the endosome to the TGN.

### Rab9a is in close proximity to HPV at early times post-infection

**Fig 2:**
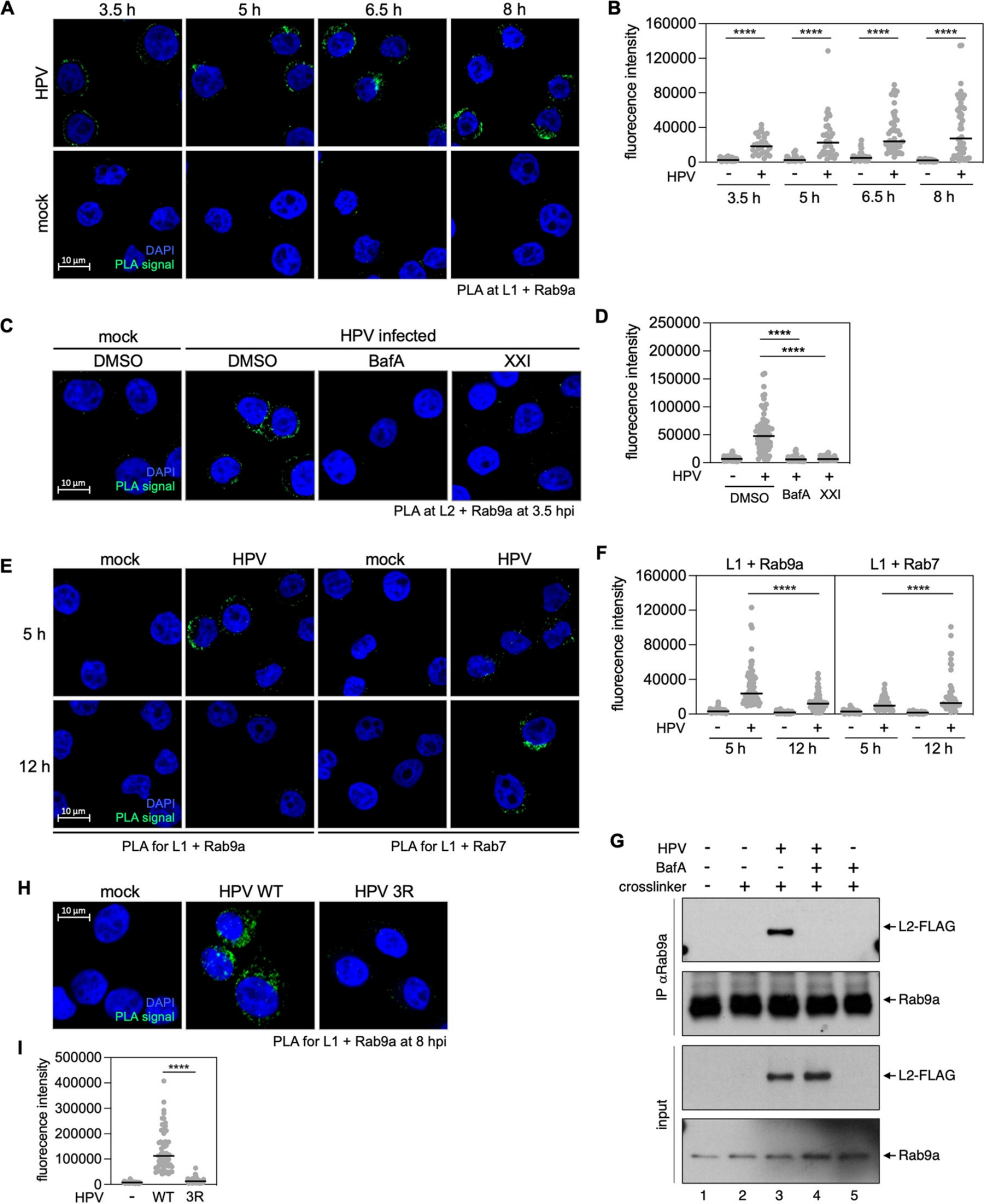
Rab9a is in proximity to HPV at early times post-infection prior to Rab7. (**A**) HeLa S3 cells were mock-infected or infected at the MOI of ~200 with HPV16 PsV L2-3XFLAG containing the HcRed reporter plasmid. At 3.5, 5, 6.5, or 8 hpi, PLA was performed with antibodies recognizing HPV L1 and Rab9a. PLA signals are green; nuclei are blue (DAPI). Similar results were obtained in two independent experiments. (**B**)The fluorescence of PLA signals was determined from multiple images obtained as in (**A**). Each dot represents an individual cell (*n*>30) and black horizontal lines indicate the mean value of the analyzed population in each group. ****, *P* < 0.0001. The graph shows results of a representative experiment. Similar results were obtained in two independent experiments. (**C**) As in (**A**) except DMSO, Bafilomycin A1 (BafA), or γ-secretase inhibitor (XXI) were added to the medium 30 min prior to infection, and PLA was performed at 3.5 hpi with antibodies recognizing FLAG (HPV L2) and Rab9a. Similar results were obtained in two independent experiments. (**D**) Images as in (**C**) were analyzed as described in (**B**). (**E**) As in (**A**) except PLA was performed at 5 and 12 hpi with antibodies recognizing HPV L1 and Rab9a or Rab7. Similar results were obtained in two independent experiments. (**F**) Images as in (**E**) were analyzed as described in (**B**). (**G**) HeLa S3 cells infected at the MOI of ~100 with HPV16 PsV L2-3XFLAG (HPV) harboring the HcRed reporter plasmid. DMSO (-) or Bafilomycin A1 (BafA, +) were added to the medium 30 min prior to infection. DSP crosslinker was added as indicated. At 8 hpi, lysates were pulled down with antibody recognizing Rab9a (IP) and subjected to Western blot analysis using antibodies recognizing FLAG, or Rab9a, together with samples not pulled down (input). (**H**) As in (**A**) at 8 hpi except HPV16 PsV containing HA-tagged (rather than FLAG-tagged) wild-type (WT) and 3R mutant L2 were used. Similar results were obtained in two independent experiments. (**I**) Images as in (**H**) were analyzed as described in (**B**).

To determine whether HPV and Rab9a are in proximity during HPV entry, we next conducted L1-Rab9a PLA at various times after infection. This analysis revealed that Rab9a is in proximity to L1 as early as 3.5 hpi, then increases and persists until at least 8 hpi (Fig 2A and 2B), showing that HPV associates with Rab9a relatively early during virus entry. HPV-Rab9a association at 3.5 hpi was also shown by PLA for L2 and Rab9a (Fig 2C and 2D).

Endosomal acidification and γ-secretase are required for HPV entry at relatively early times during infection by supporting L2 insertion into the endosome membrane [16,27,28]. γ-secretase may also act at a later step of infection [29]. When endosomal acidification was inhibited by Bafilomycin A1 (BafA) or γ-secretase was inhibited by compound XXI, the L2-Rab9a association at 3.5 hpi was abolished (Fig 2C and 2D). Similarly, Rab9a association with L1 was markedly reduced by these treatments (S3 Fig). These results indicate that endosomal acidification and γ-secretase activity are required for HPV-Rab9a association.

Rab7, which is also required for HPV entry [24], is found primarily in late endosomes [25,30], although there is evidence that Rab7 is also associated with early endosomes [31,32]. To test whether HPV associates with Rab9a prior to its association with Rab7, we performed PLA for L1 association with Rab9a and Rab7 at 5 and 12 hpi. Although there was considerable overlap at different time points, L1-Rab9a PLA signals were high at 5 hpi and displayed a significant decrease at 12 hpi, whereas L1-Rab7 PLA signals were low at 5 hpi and increased at 12 hpi (Fig 2E and 2F). Collectively, these results show that HPV comes in proximity to Rab9a prior to Rab7 in a manner dependent on endosome acidification and γ-secretase action.

### Rab9a associates with L2 upon protrusion through the endosomal membrane

To test if Rab9a is in a physical complex with L2 during HPV entry, we performed immunoprecipitation experiments in cells infected with PsV containing FLAG-tagged L2. At 6.5 hpi, cells were treated with the cell-permeable reversible cross-linker dithiobis(succinimidyl propionate) (DSP) for 30 min and lysed in buffer containing detergents (1% *n*-Dodecyl β-D-maltoside [DDM] and 0.05% Triton X-100). Anti-Rab9a antibody was then used to immunoprecipitate Rab9a and associated proteins, and the immunoprecipitates were subjected to gel electrophoresis and probed with anti-FLAG antibody. Anti-Rab9a co-immunoprecipitated the L2 protein from infected cells (Fig 2G, lanes 2 and 3), indicating that L2 and Rab9a are present in a physical complex. Inhibition of the endosomal acidification with BafA1 abolished the L2-Rab9a interaction (Fig 2G, lanes 3, 4 and 5).

Because Rab9a is cytoplasmic, it presumably binds to L2 only after L2 has protruded through the endosome membrane into the cytoplasm. This is likely to explain the inhibition of HPV-Rab9a association by BafA1 because endosome acidification is required for L2 protrusion [16,27,28]. Therefore, we tested whether L2 protrusion was required for Rab9a-HPV association. Cells were infected with an L2 mutant in which the cell-penetrating peptide sequence that drives membrane protrusion, RKRRKR, was replaced by RRR (this 3R mutant is endocytosed but impaired for membrane insertion and cytoplasmic protrusion [16,33]), and HPV-Rab9a association was assessed at 8 hpi by PLA for L1 and Rab9a. As shown in Fig 2H and 2I, the 3R mutation abolished the L1-Rab9a association, supporting the conclusion that the L2 protrusion is necessary for HPV-Rab9a association.

### Rab9a knockdown modulates the HPV-retromer interaction

**Fig 3:**
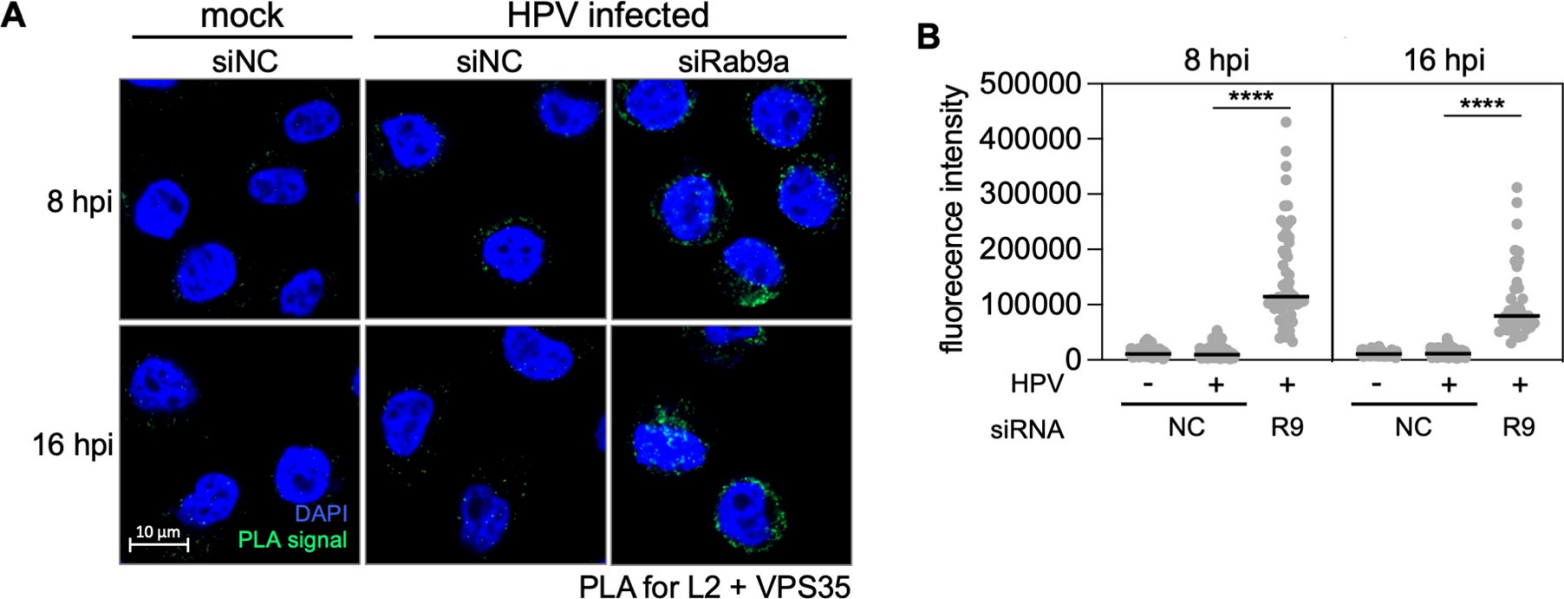
Rab9a knockdown increases HPV L2-retromer association. (**A**) HeLa S3 cells were transfected with siNC or siRab9a and infected at the MOI of ~200 with HPV16 PsV L2-3XFLAG containing the HcRed reporter plasmid. At 8 or 16 hpi, PLA was performed with antibodies recognizing FLAG and VPS35. Mock, uninfected. PLA signals are green; nuclei are blue (DAPI). Similar results were obtained in two independent experiments. (**B**) The fluorescence of PLA signals was determined from multiple images obtained as in (**A**). Each dot represents an individual cell (*n*>40) and black horizontal lines indicate the mean value of the analyzed population in each group. NC, siNC; R9, siRab9a. ****, *P* < 0.0001; ns, not significant. The graph shows results of a representative experiment. Similar results were obtained in two independent experiments.

Knockdown of retromer subunit VPS35 or VPS29 or infection with HPV16 PsV harboring retromer binding site mutations in L2 causes HPV accumulation in endosomes, indicating that the HPV-retromer interaction is required for endosome exit [13,16]. HPV accumulation in the endosome at 16 hpi was comparable in cells depleted of Rab9a and cells depleted of VPS35 (S4 Fig). Therefore, we used PLA to test whether Rab9a knockdown affected HPV interaction with retromer. Fig 3A and 3B show that the HPV-retromer interaction as measured by PLA for L2 and VPS35 at 8 and 16 hpi was markedly increased by Rab9a knockdown. L1-VPS35 PLA signals also were stronger in Rab9a knockdown cells than in control cells at both 8 and 16 hpi (S5A and S5B Fig). These data suggest that Rab9a facilitates endosome exit by regulating the HPV-retromer interaction.

Endosomal acidification and γ-secretase are required not only for HPV-Rab9a association (Fig 2) and but also L2-retromer association [27,28]. Inhibition of endosomal acidification abolished the HPV-retromer interaction at 8 hpi in the presence or absence of Rab9a (S5C and S5D Fig). Similarly, inhibition of γ-secretase also greatly reduced HPV-retromer association and largely eliminated the increase in the HPV-retromer interaction caused by Rab9a knockdown (S5C and S5D Fig). Collectively, these data show that endosomal acidification and γ-secretase activity are required for the HPV-Rab9 association and for the increased HPV-retromer association caused by Rab9a knockdown, implying that acidification and γ-secretase are required prior to Rab9a action during HPV entry.

### Rab9a knockdown increases the abundance of GTP-bound Rab7

**Fig 4:**
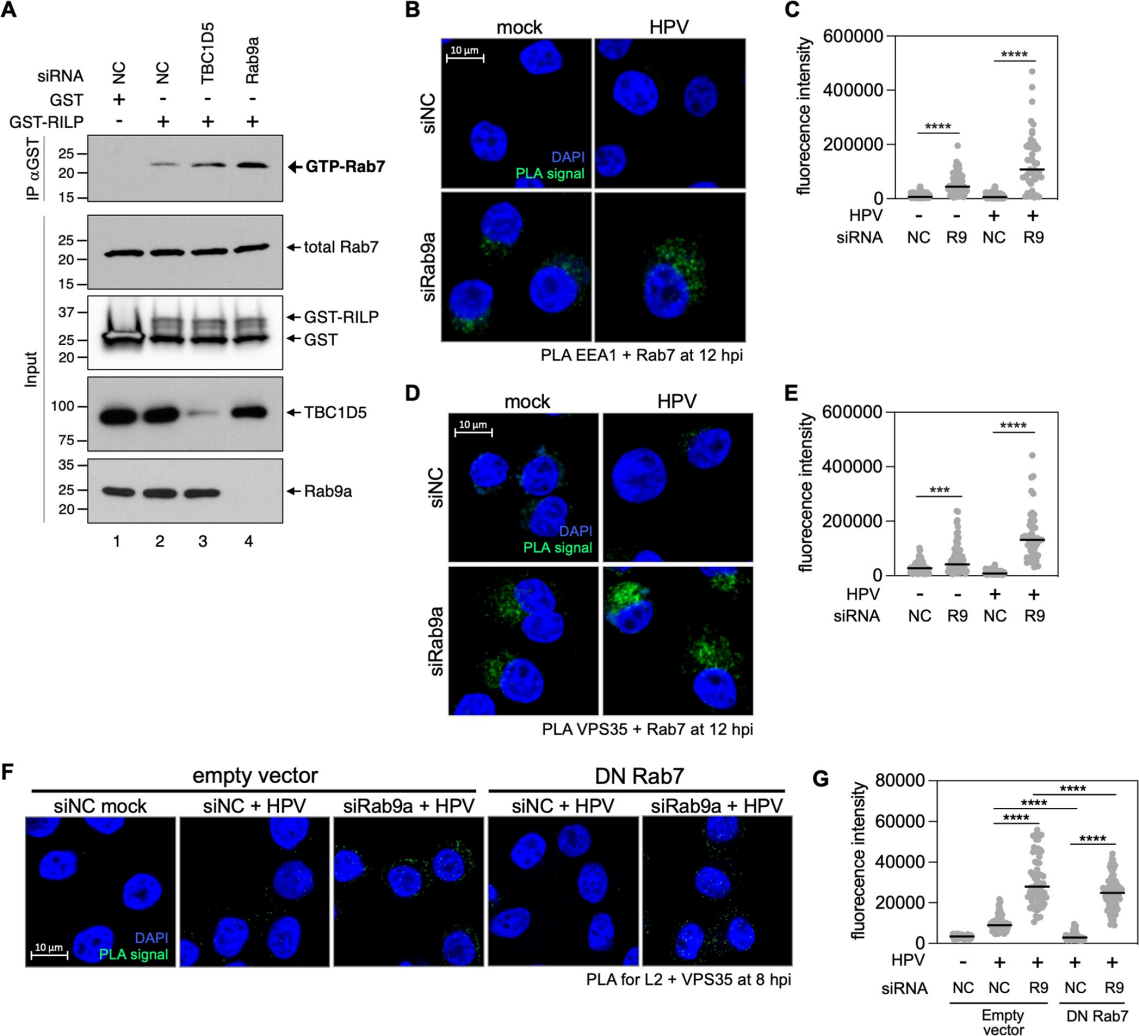
Rab9a knockdown enhances HPV-retromer association even in cells overexpressing GDP-Rab7 despite increases GTP-Rab7 abundance. (**A**) HeLa S3 cells were transfected with siNC, siRab9a or siTBC1D5. At 48 h after transfection, lysates were pulled down with GST or GST-RILP (IP) and subjected to Western blot analysis together with samples not pulled down (input) using antibodies recognizing Rab7, GST, TBC1D5, or Rab9a. Similar results were obtained in two independent experiments. (**B**) HeLa S3 cells were transfected with siNC or siRab9a and mock-infected or infected at the MOI of ~200 with HPV16 PsV L2-3XFLAG containing the HcRed reporter plasmid. At 12 hpi, PLA was performed with antibodies recognizing EEA1 and Rab7. PLA signals are green; nuclei are blue (DAPI). Similar results were obtained in two independent experiments. (**C**) The fluorescence of PLA signals was determined from multiple images obtained as in (**B**). Each dot represents an individual cell (*n*>30) and black horizontal lines indicate the mean value of the analyzed population in each group. ***, *P* < 0.001; ****, *P* < 0.0001. The graph shows results of a representative experiment. Similar results were obtained in two independent experiments. (**D**) As in (**B**) except PLA was performed at 12 hpi with antibodies recognizing VPS35 and Rab7. Similar results were obtained in two independent experiments. (**E**) Images as in (**D**) were analyzed as described in (**C**). (**F**) As in (**B**) except HeLa S3 cells stably transduced with an empty vector or a plasmid expressing dominant negative Rab7 (Rab7 DN) were used, and PLA was performed at 8 hpi with antibodies recognizing FLAG and VPS35. Similar results were obtained in two independent experiments. (**G**) Images as in (**F**) were analyzed as described in (**C**). *, *P* < 0.05.

Because knockdown of either Rab9a or Rab7 affects HPV-retromer association (Figs 3 and S5) [24], we wondered whether Rab9a affects Rab7 activity. To test whether Rab9a knockdown affected the abundance of GTP-bound Rab7, we performed pull-downs with Rab-interacting lysosomal protein (RILP), which binds GTP-bound but not GDP-bound Rab7 [34]. Purified glutathione S-transferase (GST) or a GST-RILP fusion protein was incubated with extracts from uninfected HeLa cells transfected with various siRNAs and then immunoprecipitated with anti-GST antibody and blotted with anti-Rab7 antibody. As expected, in extracts from control cells transfected with siNC, Rab7 was co-immunoprecipitated by GST-RILP but not by the negative control GST (Fig 4A, lanes 1 and 2). Knockdown of the Rab7 GTPase-activating protein (GAP) TBC1D5 caused about 2.5-fold increase in GTP-Rab7, also as expected (Fig 4A, compare lanes 2 and 3). GST-RILP pulled down approximately 4-fold more Rab7 from extracts from the cells transfected with siRab9a than from cells transfected with control siNC RNA (Fig 4A, compare lanes 2 and 4), while total Rab7 abundance did not change (Fig 4A, second panel from top). This result indicates that Rab9a knockdown increases the amount of GTP-Rab7. Moreover, Rab9a knockdown increased the localization of Rab7 to the endosome as shown by increased Rab7-EEA1 PLA signal (Fig 4B and 4C) and increased Rab7-VPS35 association as measured by PLA (Fig 4D and 4E), consistent with the ability of GTP-Rab7 –not GDP-Rab7 –to bind the retromer complex [35]. The increase of endosomal localization of Rab7 and increased Rab7-retromer interaction caused by Rab9a knockdown occurred in the absence of HPV infection and to a greater extent in cells infected with HPV (Fig 4D and 4E). We previously showed that Rab7 and HPV are in a complex at 12 hpi [24]. Here, we used PLA to demonstrate that the L1-Rab7 association in HPV infected cells was increased by Rab9a knockdown as early as 8 hpi (S6A and S6B Fig). Taken together, these data indicate that Rab9a knockdown promotes an increase of GTP-Rab7, the canonical activated form of Rab7, as well as increased HPV-retromer association.

### Rab9a knockdown increases HPV-retromer association even in the presence of dominant negative Rab7

GTP-bound Rab7 is critical for recruitment of retromer to endosomes [35]. Indeed, cells expressing dominant negative Rab7 mutant (T22N; DN Rab7), which binds only GDP and is thus unable to interact with retromer [23,35], displayed reduced HPV-retromer association at 8 and 16 hpi [23,24], showing that GTP-Rab7 supports HPV-retromer association. To test whether Rab9a regulates HPV-retromer association by decreasing GTP-Rab7, we analyzed cells knocked down for Rab9a in the presence and absence of DN Rab7. As shown in Figs 4F, 4G and S7, the PLA signals for L2-VPS35 and L1-VPS35 at 8 or 16 hpi in Rab9a knockdown cells were elevated to a similar level in the presence and absence of DN Rab7, even though cells expressing DN Rab7 contain approximately only 5% GTP-Rab7 compared to control cells (S8 Fig). Thus, when Rab9a is knocked down, GTP-Rab7 is not required for retromer-HPV association. These results suggest that Rab9a regulates retromer association independently of Rab7, consistent with the finding that HPV associates with Rab9 before it associates with Rab7.

### Excess GTP-bound Rab9a inhibits HPV infection whereas excess GDP-bound Rab9a promotes it

**Fig 5:**
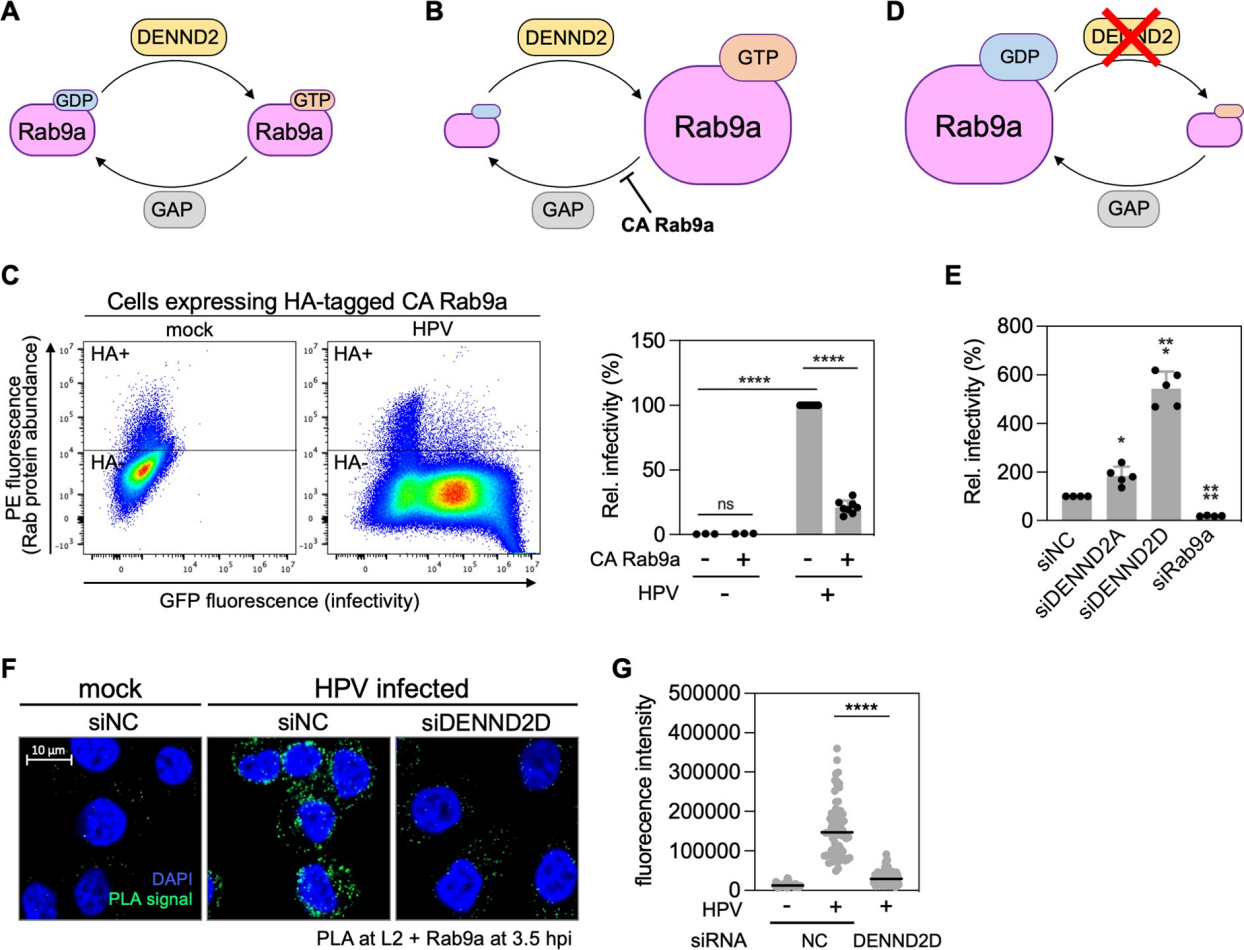
GTP-bound Rab9a inhibits HPV infection but GDP-bound Rab9a promotes it. (**A**) Schematic of Rab9a cycling between GTP-bound and GDP-bound forms. DENND2, a Rab9a GEF, converts GDP-Rab9a to GTP-Rab9a by exchanging GDP to GTP. A GTPase-activating protein (GAP) hydrolyzing GTP-Rab9a is yet to be identified. (**B**) Constitutively active (CA) Rab9a mutant is locked in GTP-bound form; thus, cells expressing CA Rab9a accumulate GTP-Rab9a. (**C**) 293TT cells were transfected with a plasmid expressing HA-tagged CA Rab9a. At 48 h after transfection, cells were infected at the MOI of ~2 with HPV16 PsV L2-3XFLAG containing the GFP reporter plasmid. At 48 hpi, samples were stained using PE-conjugated antibody recognizing HA, and flow cytometry was used to determine PE and GFP fluorescence. Cells expressing HA-tagged CA Rab9a were marked as HA+, and those not expressing such proteins were marked as HA-. Representative dot blots are shown in left panels. On right, data from multiple repeats are shown as percent relative infectivity normalized to populations not expressing Rab9a CA (set at 100%). Each dot shows the result of an individual experiment. Bars and error bars show mean and standard deviation (SD), respectively. ****, *P* < 0.0001; ns, not significant. (**D**) Knockdown of DENND2 inhibits the conversion of GDP-Rab9a to GTP-Rab9a, thereby accumulating GDP-Rab9a. (**E**) HeLa S3 cells transfected with siNC, siDENND2A, siDENND2D, or siRab9a were infected at the MOI of ~0.2–0.4 with HPV16 PsV L2-3XFLAG (HPV) harboring the GFP reporter plasmid. At 48 hpi, GFP fluorescence was determined by flow cytometry. The results were shown in percent relative infectivity normalized to the siNC treated cells (set at 100%). Each dot shows the result of an individual experiment. Bars and error bars show mean and SD, respectively. *, *P* < 0.05; ***, *P* < 0.001; ****, *P* < 0.0001. (**F**) HeLa S3 cells transfected with siNC or siDENND2D were infected at the MOI of ~200 with HPV16 PsV L2-3XFLAG containing the HcRed reporter plasmid. At 3.5 hpi, PLA was performed with antibodies recognizing FLAG and Rab9a. Mock, uninfected. PLA signals are green; nuclei are blue (DAPI). Similar results were obtained in two independent experiments. (**G**) The fluorescence of PLA signals was determined from multiple images obtained as in (**F**). Each dot represents an individual cell (*n*>40), and horizontal lines indicate the mean value of the analyzed population in each group. ****, *P* < 0.0001. The graph shows results of a representative experiment.

The GTP-bound form of Rab proteins typically promotes cargo trafficking [1]. To determine whether GTP-Rab9a is necessary for HPV infection, we transiently introduced constitutively active (CA) HA-tagged Rab9a mutant (Q66L) into 293TT cells. Cells expressing CA Rab9a are predicted to accumulate GTP-Rab9a and have less GDP-Rab9a, compared to normal cells (Fig 5A and 5B). 48 h after transfection, the cells were infected with HPV16 PsV. Forty-eight hpi we used flow cytometry to quantify GFP fluorescence as a measure of infection and anti-HA staining to document successful expression of the mutant Rab9a protein. This experimental design allows us to compare HPV infection in the presence or absence of CA Rab9a (i.e., in HA-positive and HA-negative cells, respectively) in the same cell population (Fig 5C). Contrary to our expectation, cells expressing CA Rab9a with presumably an increased GTP-Rab9a to GDP-Rab9a ratio reduced HPV infection (Fig 5C), indicating that excess GTP-Rab9a impairs HPV infection.

Next, we determined the effect of excess GDP-Rab9a on HPV infection. We were not able to use DN Rab9a mutant (S21N) for this experiment because we were unable to generate a large enough number of cells expressing DN Rab9a. As an alternative approach to generate cells with excess GDP-Rab9a, we knocked down two isoforms of DENND2 (a guanine nucleotide exchange factor (GEF) for Rab9a), which should cause accumulation of GDP-bound Rab9a (Fig 5D). DENND2D knockdown resulted in 5-6-fold higher HPV infectivity in HeLa cells compared to cells transfected with the control siRNA (Fig 5E), and DENND2A knockdown resulted in ~2-fold higher infectivity (Fig 5E). This difference is consistent with the higher Rab9a GDP-exchanging activity of DENND2D compared to DENND2A [36]. The DENND2D knockdown also increased HPV16 PsV infection in HaCaT cells (S9A and S9B Fig). Similarly, HPV18 and HPV5 PsV also showed increased infection in DENND2D knockdown HeLa cells (S9C Fig). Thus, HPV infection was inhibited by GTP-bound CA Rab9a and stimulated by excess GDP-Rab9a.

Finally, we tested which form of Rab9a interacts with L2. We used PLA to examine L2-Rab9a interaction in control and DENND2D knockdown cells. L2-Rab9a PLA signals at 3.5 hpi were lower in DENND2D knockdown cells containing excess GDP-Rab9a than in control cells (Fig 5F and 5G), suggesting that L2 primarily interacts with GTP-Rab9a.

Collectively, these data indicate that an increased ratio of GDP-Rab9a to GTP-Rab9a disrupts the L2-Rab9a association and promotes HPV entry, whereas an increased ratio of GTP-Rab9a to GDP-Rab9a increases the interaction and impairs entry.

### Trafficking of cellular cargo is promoted by GTP-Rab9a

**Fig 6:**
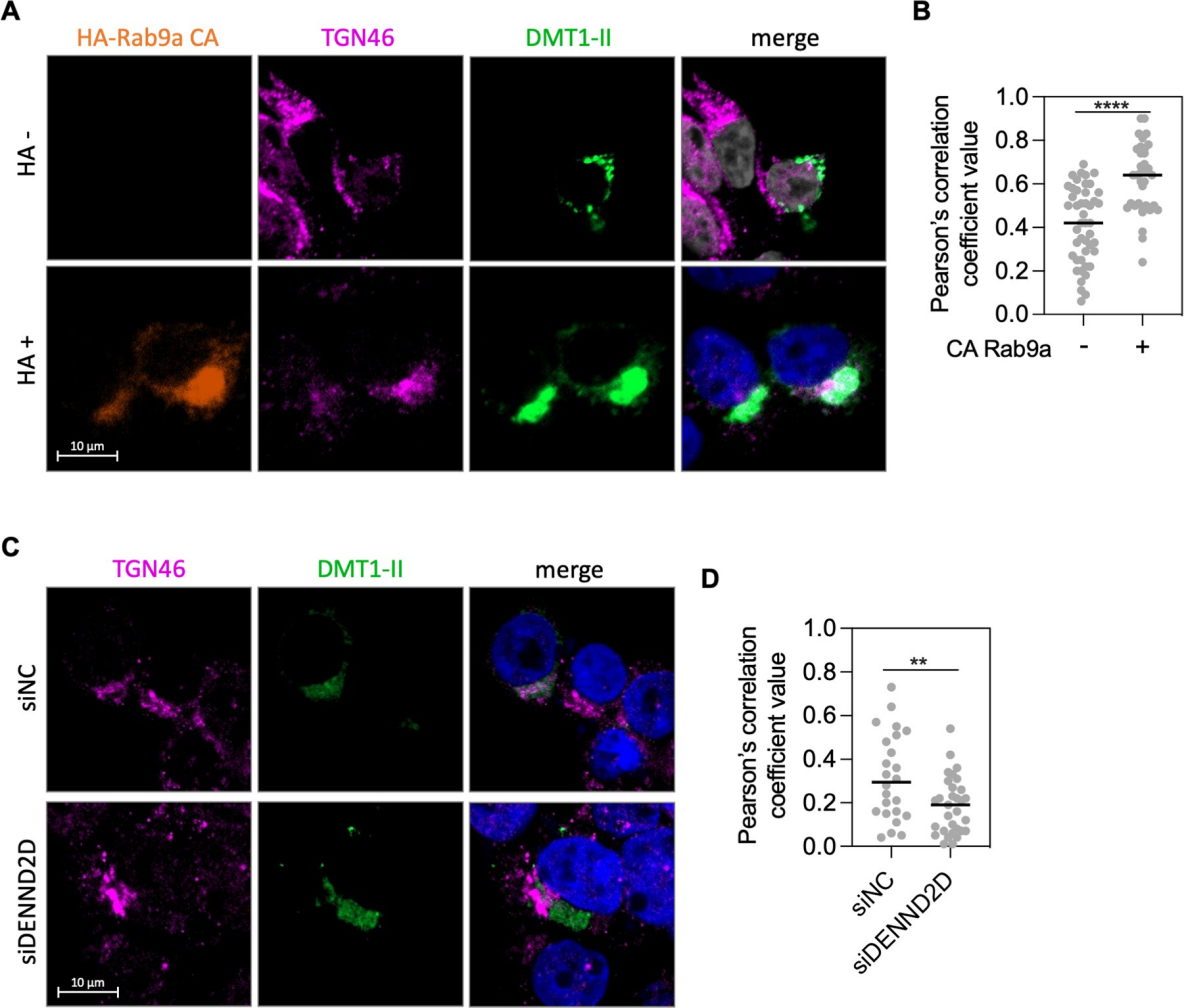
GTP-Rab9a promotes trafficking of DMT1-II. (**A**) 293TT cells were transfected with a plasmid expressing HA-Rab9a CA, followed by transfection of a DMT1-II expressing plasmid 24 h after the first transfection. Expression of HA-Rab9a CA was determined by anti-HA staining, which distinguishes cells expressing HA-Rab9a CA (+) or those not expressing it (-). DMT1-II and TGN46 were stained using antibodies recognizing GFP and TGN46, respectively. Immunofluorescence (IF) images were shown; HA-Rab9a CA, orange; TGN46, magenta; DMT1-II, green; nuclei (DAPI), blue. Merged image shows TGN46 and DMT1-II with overlap colored white. Similar results were obtained in three independent experiments. (**B**) Pearson’s correlation coefficient values for TGN46 and DMT1-II colocalization in those cells are shown. Each dot represents an individual cell (*n*>25), and horizontal lines indicate the mean value of the analyzed population in each group. ****, *P* < 0.0001. The graph shows results of a representative experiment. Similar results were obtained in three independent experiments. (**C**) cells were transfected with siNC or siDENND2D, followed by transfection of a DMT1-II expressing plasmid 48 h after the first transfection and stained for TGN46 and DMT1-II as in (**A**). Similar results were obtained in two independent experiments. (**D**) Images as in (**C**) were analyzed as described in (**B**). **, *P* < 0.01.

**Fig 7:**
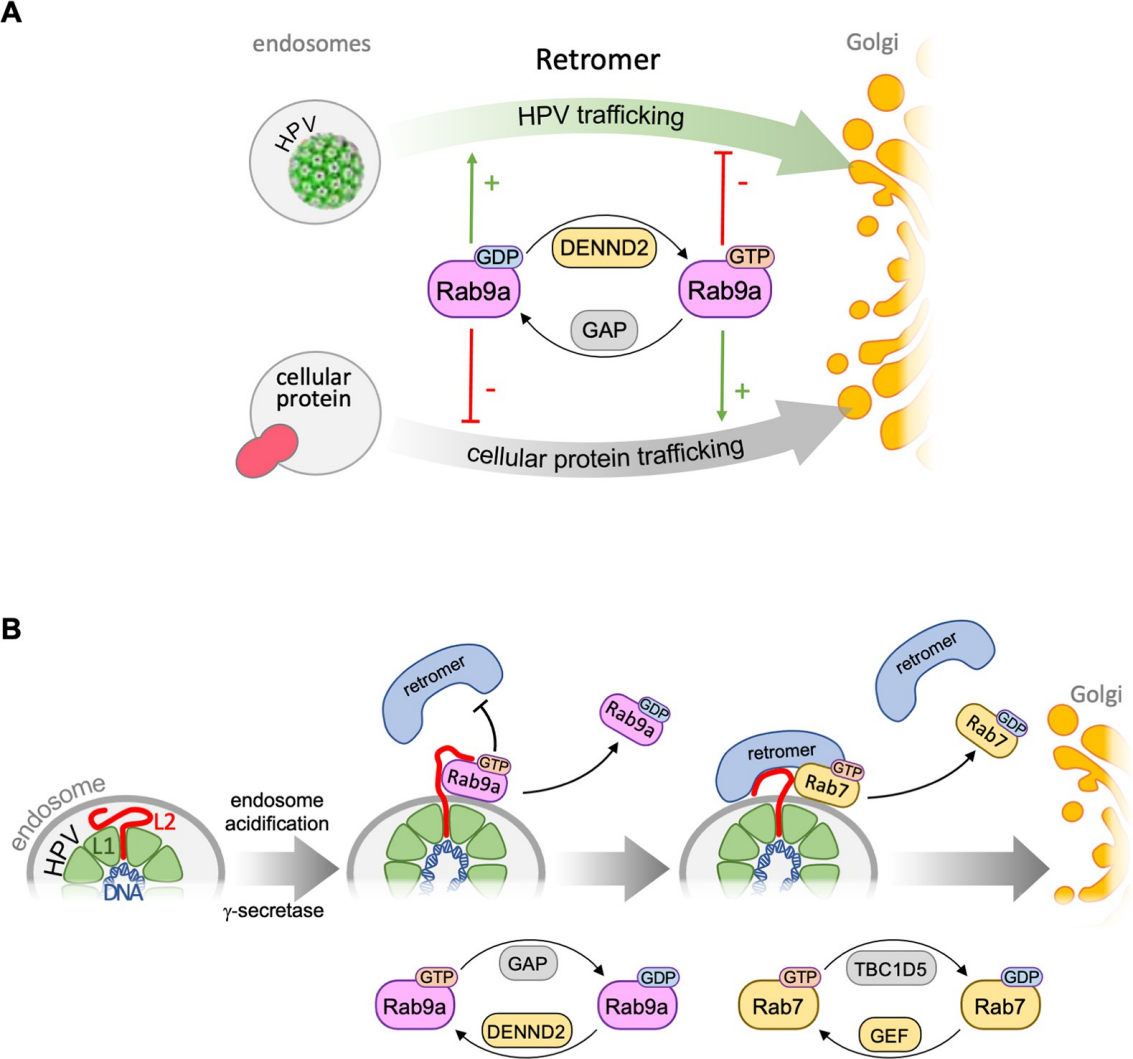
GDP-bound Rab9a promotes HPV entry by supporting retromer-mediated retrograde HPV trafficking. (**A**) Retrograde transport of cellular protein(s), such as CI-MPR and DMT1-II, from endosome to Golgi requires GTP-bound Rab9a and is impaired by GDP-bound Rab9a, as is the case for other known Rab proteins. Surprisingly, however, HPV entry is stimulated by GDP-bound Rab9a, previously thought to be an inactive form, whereas the GTP-bound Rab9a inhibits HPV entry. DENND2 impairs HPV entry by decreasing GDP-Rab9a and increasing GTP-Rab9a. (**B**) Internalized HPV undergoes retrograde trafficking by sequentially associating with Rab9a and Rab7 during virus entry, which is enabled by hydrolysis of GTP bound to each Rab protein. This model suggests that HPV is “handed over” from Rab9a to Rab7 during trafficking (please see the text for detailed information).

Rab9a is required for the retrograde trafficking of cellular proteins [25]. Unlike HPV trafficking as described above, endosome to TGN transport of cation-independent mannose-6-phosphate receptor (CI-MPR) is impaired by the increase of GDP-Rab9a in cells expressing Rab9a DN or depleted of DENND2 [36,37], indicating that CI-MRP trafficking requires GTP-Rab9a. To investigate further the role of GTP-Rab9a action in cellular protein cargo trafficking in our cell system, we used immunofluorescence to monitor the effect of modulating Rab9a on a different cellular retromer cargo protein, divalent metal transporter 1 isoform II (DMT1-II). Consistent with previously reported phenotypes on CI-MPR trafficking [36,37], CA Rab9a increased the co-localization of DMT1-II and TGN46 in 293TT cells (Fig 6A and 6B), whereas DENND2D knockdown decreased co-localization of DMT1-II and TGN46 compared to the control cells (Fig 6C and 6D). Increased co-localization of DMT1-II and TGN46 in cells expressing CA Rab9a was also observed when cells were infected with HPV16 PsV (S10 Fig). These results suggest that, in contrast to HPV trafficking, increased GTP-Rab9a to GDP-Rab9a ratio promotes trafficking of DMT1-II from endosome to TGN, whereas increased GDP-Rab9a to GTP-Rab9a ratio inhibits DMT1-II trafficking. These data indicate that Rab9a acts differently on cellular protein transport and HPV trafficking (Fig 7A).

## Discussion

Rab GTPases play key regulatory roles in intracellular vesicular trafficking [1,2,4–6]. Like other small GTPases, Rab proteins exist in two forms, an active GTP-bound state necessary for trafficking of cellular cargo and an inactive GDP-bound state [1,3]. Accordingly, GTP-Rab9a supports the transport of cellular cargos such as CI-MPR and DMT1-II (Fig 6) [36,37]. Surprisingly, however, we show here that HPV trafficking during entry is impaired by excess GTP-Rab9a and stimulated by excess GDP-Rab9a (Figs 5A–5E and S9). These findings indicate that HPV and cellular proteins utilize the Rab9a host trafficking machinery in distinct ways during intracellular trafficking (Fig 7A).

Retrograde trafficking of HPV from endosome to TGN requires the interaction of retromer with L2, followed by retromer-L2 dissociation [12,13]. The findings reported here as well as our previously published work indicate that the association of retromer to HPV-containing vesicles is regulated by different Rab proteins at different times post infection (Fig 7B). We previously reported that GTP-Rab7 recruits retromer to HPV at the endosome membrane during HPV entry and that TBC1D5-stimulated hydrolysis of GTP-Rab7 to GDP-Rab7 allows dissociation of HPV from retromer [24], a step required for trafficking to proceed [1,3,6,12,15]. Thus, both GTP-Rab7 and GDP-Rab7 are required for HPV infection. In contrast, the GTP- and GDP-bound forms of Rab9a have opposing effects on HPV infection, with excess GDP-Rab9a supporting infection and excess GTP-Rab9a inhibiting it. Furthermore, Rab9a associates with HPV prior to Rab7 and the HPV-retromer interaction is enhanced when Rab9a is knocked down. In contrast to HPV entry, other viruses appear to be dependent on GTP-bound Rab proteins [4,38,39]. Although the trafficking of the Hepatitis C Virus (HCV) is mediated by GTP-bound Rab proteins [39], the assembly of HCV requires GDP-bound Rab32 [40]. These observations suggest viruses may exploit a variety of non-canonical roles of Rab proteins.

The preferential association of L2 with GTP-Rab9a revealed by DENND2 knockdown (Fig 5F and 5G) is consistent with the typical behavior of Rab proteins, which bind their partners when GTP bound [3]. However, excess GTP-bound Rab9a inhibits HPV trafficking, whereas excess GDP-Rab9a, which stimulates trafficking, is relatively impaired for L2 binding. We believe that this situation can be explained because HPV L2-Rab9a association occurs early during infection and is transient. According to this model, L2 and GTP-Rab9a associate early in infection in a manner dependent of endosome acidification and γ-secretase activity. Hydrolysis of GTP bound to Rab9a allows GDP-Rab9a to dissociate from L2, whereupon GTP-Rab7 associates with L2 and productively recruits retromer to HPV at the endosome (Fig 7B). Hydrolysis of GTP bound to Rab7 then allows Rab7 and retromer to dissociate from HPV in order for trafficking to proceed. Because GTP-Rab9a appears to bind L2 better than GDP-Rab9a, constitutively active GTP-Rab9a may inhibit HPV entry by interfering with the transition of L2 from binding Rab9a to binding Rab7. The ordered transient interactions with different Rab proteins may allow L2 to sequentially engage multiple host factors at the appropriate time during virus entry.

Because retromer-HPV dissociation is required for endosome exit [24], it is likely that the increased HPV-retromer association observed in Rab9a knockdown cells is responsible for endosomal accumulation of HPV in these cells. Consistent with this idea, Rab9a knockdown increased HPV-retromer association by 8 hpi, before HPV accumulates in endosomes (Figs 1, 3 and S5). Given our previous finding that GTP-Rab7 increases HPV-retromer association [24], the increased GTP-Rab7 abundance in Rab9a knockdown cells is predicted to prevent HPV-retromer dissociation. However, Rab9a knockdown increases retromer-HPV interaction even in cells expressing DN Rab7 (Figs 4F, 4G and S7), although in the absence of Rab9a knockdown, DN Rab7 inhibits HPV-retromer association (Fig 4G) [24]. This implies that the enhanced HPV-retromer association in cells depleted of Rab9a is not mediated by increased GTP-Rab7. Indeed, the increased retromer-HPV association in the Rab9a knockdown cells even if they express DN Rab7 implies the existence of a Rab7-independent mechanism that non-productively recruits retromer to HPV in cells with reduced levels of Rab9a. For example, it is possible that retromer is recruited to the HPV-containing endosomal vesicle via sorting nexins (SNXs) in the absence of Rab9a and active Rab7. In support of this notion, *in vitro* studies showed that SNX3 in the absence of Rab proteins increased interaction of retromer with a peptide derived from L2 [41].

Because both Rab9a and Rab7 interact with retromer [35,42] and Rab9a knockdown increased Rab7 association with retromer (Fig 4D and 4E), Rab9a bound to retromer may directly interfere with the ability of Rab7 to bind retromer and HPV. Competitive binding of Rab9a and Rab7 to retromer may allow Rab9a to delay Rab7 binding to HPV and/or retromer until Rab9a dissociates.

A previous report claimed that GDP-Rab9a inhibits HPV infection [15], in contrast to our findings. Day et al. [15] used DsRed-tagged DN Rab9a expressed in 293TT cells and used flow cytometry to assess fluorescence in the entire transfected cell population. They did not test the effect of expression of constitutively active Rab9a. In contrast, we assessed the effects of HA-tagged CA Rab9a and used flow cytometry to measure reporter protein fluorescence and mutant Rab9a expression in individual cells in the same cell population (containing cells that expressed CA Rab9a and those that did not), which showed that HPV infectivity is decreased in cells expressing CA Rab9a. Furthermore, in HeLa cells, a more physiologically relevant cell type, DENND2 knockdown increases GDP-Rab9a and stimulates HPV infectivity (Figs 5E and S9). These multiple lines of evidence support our conclusion that excess GDP-Rab9a promotes HPV infection whereas excess GTP-Rab9a impairs it. Nevertheless, these different findings raise the possibility that various forms of Rab9a may support HPV infection in different ways depending on subtle differences in assay conditions.

Our findings reveal that HPV and cellular protein cargos employ host trafficking machinery in distinct ways. An excess of the canonically active GTP-Rab9a and GTP-Rab7 support retrograde trafficking of cellular proteins [24,36,37] (Figs 6 and S10), whereas an excess of the canonically inactive GDP-Rab9a and cycling between GTP- and GDP-bound Rab7 support HPV entry. If the cellular cargo trafficking machinery is limiting and HPV uses these Rab proteins in the same way as the cellular protein cargo, the HPV entry process might compete with cellular protein trafficking and interfere with this process and trigger a host response that would inhibit virus infection. Alternatively, if cellular cargo out-competes the virus, virus entry would be impaired. Thus, HPV may have evolved to use the cellular trafficking system in a distinct way from cellular protein cargo to avoid competition for the entry machinery and ensure successful entry. Other viruses compete with host protein transport to evade the host immune response. For example, Middle East respiratory syndrome coronavirus and Ebola virus prevent the nuclear import of host transcription factors by competing with importins that normally facilitate nuclear import of these factors [43,44]. The use of distinct trafficking strategies by HPV compared to cellular cargo may allow the development of therapeutic approaches to control HPV infection by trapping Rab9a in its GTP-bound form to block HPV entry while permitting cellular cargo to traffic.

## Materials and methods

### Cell lines

HeLa S3 cells were purchased from American Type Culture Collection (ATCC). HaCaT cells were purchased from AddexBio Technologies. 293TT cells were generated by introducing SV40 Large T antigen cDNA into HEK293T cells to increase Large T antigen expression and obtained from Christopher Buck (NIH). All cell lines were cultured at 37°C and 5% CO_2_ in Dulbecco’s modified Eagle’s medium (DMEM) supplemented with 20 mM HEPES, 10% fetal bovine serum (FBS), L-glutamine, and 100 units/mL penicillin streptomycin (DMEM10). Cell identity was confirmed by ATCC cell authentication service.

### Production of HPV pseudovirus (PsV)

HPV16 PsVs were produced by co-transfecting 293TT cells with wild-type p16SheLL-3XFLAG tag [14] together with pCAG-HcRed (Addgene #11152) or pCINeo-GFP [obtained from C. Buck] using polyethyleneimine (MilliporeSigma). For HPV18 and HPV5, p18SheLL-HA tag and p5SheLL were used, respectively. PsVs were purified by density gradient centrifugation in OptiPrep (MilliporeSigma) as described [45]. For comparing wild-type and 3R mutant, p16SheLL-HA WT and 3R mutant [16] were used. Briefly, cells were washed with Dulbecco’s Phosphate Buffered Saline (DPBS) at 24 h post transfection, incubated in DMEM10, and collected 72 h post transfection in siliconized tubes. The cells were then incubated in lysis buffer (DPBS with 0.5% Triton X-100, 10 mM MgCl_2_, 5 mM CaCl_2_, 100 μg/mL RNAse A (Qiagen)) overnight at 37°C in a water bath to allow capsid maturation. The lysates containing matured PsVs were loaded on an OptiPrep gradient that had been stabilized at least 1 h and centrifuged at 50k x g for 3.5–4 h at 4°C in a SW-55Ti rotor (Beckman). Fractions were collected in siliconized tubes and subjected to SDS-PAGE followed by Coomassie blue staining to assess the abundance of L1 and L2 proteins. Peak fractions were pooled, aliquoted, and stored at -80°C.

### Determining HPV PsV infectivity

0.37x10^5^ HeLa S3 cells per well were plated in 24-well plates ~48 h prior to infection. Approximately 6 h later, cells were transfected with 6.7 nM of indicated siRNAs (S1 Table) using Lipofectamine RNAiMAX (Invitrogen) according to manufacturer’s protocol. Non-targeting siRNA (S1 Table) was used as a negative control. 40–48 h after transfection, cells were infected with PsVs at an infectious MOI of ~5 or ~0.5 in unmodified HeLa cells. At 48 hpi, infectivity was determined by using flow cytometry to measure fluorescence intensity produced from expression of the reporter gene. Relative percent infectivity was determined by normalizing mean fluorescence intensity of samples transfected with experimental siRNA to that of the cells transfected with control siRNA, which was set at 100%.

### Western blot analysis

siRNA-treated cells were lysed using ice-cold 1X Radioimmunoprecipitation assay (RIPA) [50 mM Tris (pH 7.4), 150 mM NaCl, 1% Nonidet P-40, 1% sodium deoxycholate, 0.1% sodium dodecyl sulfate, 1 mM Ethylenediaminetetraacetic acid] buffer supplemented with 1X HALT protease inhibitor cocktail (Pierce) for 15 min at 4°C. After centrifugation at 14,000 rpm for 15 min at 4°C, the protein concentration in the supernatant was determined by Bicinchoninic acid (BCA) protein assay (Pierce). After normalization for protein amounts, the supernatant was mixed with 4X Laemmli dye (Bio-rad) supplemented with 10% 2-mercaptoethanol and incubated in a water bath for 7 min at 100°C. Samples were then separated by SDS-PAGE (4–12% gel) (Bio-rad) and analyzed by Western blotting using antibodies recognizing Rab9a (Thermo Fisher, 11420-1-AP, 1:1,000), Rab7 (Cell Signaling Technology, 9367, 1:1,000), TBC1D5 (Abcam, ab203896, 1:1,000), pan actin (Cell Signaling Technology, 4968, 1:2,000), FLAG (Sigma, A8592, 1:1,000) or GST (Santa Cruz, sc-138, 1:2,000). Secondary horseradish peroxidase (HRP)-conjugated antisera recognizing rabbit or mouse antibodies as appropriate (Jackson ImmunoResearch, 711-035-152, 115-035-146) were used at 1:5,000 dilution in 5% non-fat milk unless specified otherwise. The blots were developed with SuperSignal West Pico or Femto Chemiluminescent substrate (Pierce) and were visualized by using a FluorChem Imager (Bio-technne, FE0685) or film processor (Fujifilm).

### Proximity ligation assay (PLA)

0.35x10^5^ HeLa S3 cells per well were plated in 24-well plates containing glass coverslips 48 h prior to infection. Approximately 6 h later, cells were transfected with 6.7 nM of indicated siRNA (S1 Table) as described above. At 40–48 h after transfection, cells were infected with PsVs at MOI of ~200 in unmodified HeLa cells. As indicated, DMSO (0.2% as final concentration), 100 nM BafA, or 2 μM XXI were added to the medium 30 min prior to infection. At indicated times post-infection, cells were fixed with 4% paraformaldehyde (Electron Microscopy Sciences) at room temperature (RT) for 12 min, permeabilized with 1% Saponin (Sigma-Aldrich) at RT for 35–40 min, and blocked using DMEM10 at RT for 1–1.5 h. Cells were then incubated overnight at 4°C with a pair of mouse and rabbit antibodies: a mouse antibody recognizing L1 (BD Biosciences, 554171, 1:1,000 when used with anti-TGN46, 1:200 when used with other antibodies), FLAG or HA on the L2 protein (anti-FLAG, SIGMA, F1804, 1:1000; anti-HA, BioLegend, 901513, 1:200), or cellular proteins (anti-EEA1, BD Biosciences, 610457, 1:100; anti-VPS35, Abcam, ab57632, 1:200; anti-LAMP1, Abcam, ab25630, 1:150); and a rabbit antibody recognizing cellular proteins or epitope tags (anti-TGN46, Abcam, ab50595, 1:600; anti-EEA1, Cell Signaling Technology, 2411, 1:75; anti-VPS35, Abcam, ab157220, 1:200; anti-Rab9a, Thermo Fisher, 11420-1-AP, 1:200; anti-Rab7, Cell Signaling Technology, 9367S, 1:200; anti-HA, Cell Signaling Technology, 3724S, 1:200; anti-FLAG, Cell Signaling Technology, 14793, 1:150). PLA was performed with Duolink reagents (Sigma Aldrich) according to the manufacturer’s instructions as described [46]. Briefly, cells were incubated in a humidified chamber at 37°C with a pair of PLA antibody (mouse and rabbit) probes for 75 min, with ligation mixture for 45 min, and then with amplification mixture for 3 h, followed by series of washes. Nuclei were stained with 4,6-diamidino-2-phenylindole (DAPI). Cellular fluorescence was imaged using the Zeiss LSM980 confocal microscope. Images were processed using a Zeiss Zen software version 3.1 and quantified using Image J Fiji version 2.3.0/1.53f.

### Co-immunoprecipitation of Rab9a and L2

7x10^5^ HeLa S3 cells per pate were plated in 6 cm^2^ plates then infected with PsVs at the MOI of ~100. As indicated, 100 nM BafA was added 30 min prior to infection (non-treated control cells were treated with DMSO alone (0.1–0.2% as final concentration)). At 6.5 hpi, cells were washed twice with DPBS and cross-linked with 1.5 mM DSP in DPBS for 30 min at RT. The reaction was then quenched with 100 mM Tris HCl (pH 7.4) for 15 min at RT. The cells were then washed three times with ice cold DPBS and lysed in 500 μL of lysis buffer (20 mM HEPES [pH 7.4], 50 mM NaCl, 5 mM MgCl_2_, 1% *n*-Dodecyl β-D-maltoside (DDM) and 0.05% Triton X-100) supplemented with 1 X HALT protease and phosphatase inhibitor cocktail (Thermo Fisher). The lysate was centrifuged at 14,000 rpm for 20 min at 4°C and supernatant was transferred to new tubes and normalized for total protein amounts determined by BCA assay. 50 μL of supernatant was reserved for input samples. The remainder of the supernatant was incubated with 6 μl of anti-Rab9a antibodies overnight at 4°C, then incubated with 40 μl of protein G magnetic beads (Thermo Fisher Scientific) for one hour at room temperature. Bound proteins were collected with magnet, washed three times with the lysis buffer, and eluted with 50 μL of 2X Laemmli sample buffer (Bio-Rad) containing 5% 2-mercaptoethanol to reverse the cross-link at 95°C, followed by SDS-PAGE and Western blot analysis carried out as described above with the following modifications: anti-FLAG antibody diluted in 3% bovine serum albumin (BSA) was pre-incubated overnight at 4°C with a membrane containing total lysate from uninfected cells, then antibody was transferred into the membrane containing immunoprecipitated samples.

### RILP pull down assay

Plasmids expressing GST-RILP (obtained from Christopher Burd, Yale University) or GST alone (from pGEX KG vector; Addgene #77103) were transformed into *Escherichia coli* strain BL21(DE3). Bacterial cultures at OD_600_ of 0.5–0.6 were induced by 0.5 mM isopropyl β-D-1-thiogalactopyranoside (IPTG) at 22°C for 5 h. Bacteria were harvested, washed with Tris-Buffered Saline (TBS) (50 mM Tris [pH 8.0], 150 mM NaCl), and lysed with B-per lysis buffer (Thermo Scientific) supplemented with 1X HALT protease inhibitor cocktail (Pierce). GST-RILP or GST proteins were purified by using a pre-equilibrated slurry of glutathione beads (Thermo Scientific, #16100) in TBS containing 1mM MgCl_2_ and washed three times in the same buffer containing 0.05% Triton X-100. Purified proteins were eluted from the beads with reduced 20mM glutathione in TBS containing 1mM MgCl_2_ and dialyzed into HEPES buffer (20 mM HEPES [pH 7.4], 50 mM NaCl, 5 mM MgCl_2_, 1 mM dithiothreitol (DTT)) using Slide-A-Lyzer dialysis Cassette. Protein amounts were quantified with BSA standards separated by SDS-PAGE followed by Coomassie blue staining.

2x10^5^ HeLa S3 cells per plate were plated in 6 cm^2^ plates and transfected with 6.7 nM of indicated siRNAs (S1 Table) as described above. 40–48 h after transfection, cells were infected with PsVs at MOI of ~150 in unmodified HeLa cells. At 12 hpi, cells were lysed using 400 μL ice-cold lysis buffer (HEPES buffer containing 0.15% Triton X-100) supplemented with 1X HALT protease and phosphatase inhibitor cocktail. After centrifugation at 14,000 rpm for 20 min at 4°C, the protein concentration in the supernatant was determined by BCA protein assay (Pierce). After normalization for protein amounts, the supernatant was incubated with 15 μg of purified GST or GST-RILP proteins at 4°C for 2 h in 500 μl of lysis buffer. 40 μL of mixtures were taken for input samples. Then 40 μL of pre-equilibrated (using lysis buffer) slurry of glutathione beads were added and the mixtures were further incubated at 4°C for 3 h, followed by three washes in lysis buffer. Bound proteins were eluted with 50 μL of 2X Laemmli sample buffer containing 5% 2-mercaptoethanol at 95°C for 5 min. Input and eluted samples were separated by SDS-PAGE, and Western blot analysis was carried out as described above.

### Construction of plasmids

Plasmids expressing HA-tagged Rab9a CA or DN variants were constructed as follows: CA and DN mutant *Rab9a* genes were amplified from JB84 (Addgene #128908) and DsRed-rab9 DN (Addgene, Plasmid #12676), respectively, using primers HA-Rab9a-BamHI-F. (TGA ACC GTC AGA TCG CCT GGA GAA GGA TCC ATG TAC CCA TAC GAC GTT CCA GAT TAC GCT GCA GGA AAA TCA TCA CTT TTT AAA GTA) and Rab9a-EcoRI-R (GAA AAG CGC CTC CCC TAC CCG GTA GAA TTC TCA ACA GCA AGA TGA GCT AGG CTT GGG CTT), then introduced between BamHI and EcoRI sites of pRetroX-Tight-Pur vector (Takara, 632105). Resulting plasmids containing the desired mutation were confirmed by DNA sequencing.

### Determining HPV PsV infectivity in cells transiently expressing Rab9a CA

0.35x10^5^ 293TT cells per plate were plated in 24-well plates 16–20 h prior to transfection of plasmids encoding HA-tagged CA Rab9a or the empty vector as control (Takara, 632105). 48 h after transfection, cells were infected with PsVs at MOI of ~1 in unmodified HeLa cells. 48 hpi, cells were fixed with 4% paraformaldehyde, permeabilized with 1% Saponin, and blocked with 3% BSA. Cells were then stained using PE-conjugated antibodies recognizing HA (MACS Molecular, 130-120-717, 1:1000), followed by three to four times of washes using DPBS containing 0.1% Tween-20. HA-tagged Rab9a CA or DN protein abundance and HPV PsV infectivity were determined by flow cytometry measuring fluorescence intensity produced by PE-labeled proteins and expressed GFP simultaneously.

### Cellular protein DMT1-II trafficking assay

To analyze cells overexpressing Rab9a CA, 0.3x10^5^ 293TT cells per well were plated in 24-well plates containing glass coverslips 16–20 h prior to transfection of 2 μg plasmid encoding HA-tagged CA Rab9a, followed by transfection of 1 μg plasmid encoding DMT1-II-GFP the next day. 24 h after the second transfection, cells were processed as described below. Where indicated, cells were infected at MOI of ~100 with HPV16 PsV at 8 h after the second transfection. To examine cells depleted for DENND2D, 0.3x10^5^ 293TT cells per plate were plated in 24-well plate containing glass coverslips and transfected with 6.7 nM of indicated siRNAs (S1 Table) as described above. 40–48 h post siRNA-transfection, cells were then transfected with 1 μg plasmid encoding DMT1-II-GFP. 24 h after the second transfection, samples were fixed with 4% paraformaldehyde at RT for 10 min, permeabilized with 1% Saponin at RT for 35–40 min, and blocked using DMEM10 at RT for 1–1.5 h. Cells were then incubated overnight at 4°C with a mouse antibody recognizing GFP (Santa Cruz, sc-9996, 1:300) and a rabbit antibody recognizing TGN46 (Abcam, ab50595, 1:250). A rat antibody recognizing HA was used (Roche, 11867423001, 1:250) to visualize HA-tagged CA Rab9a. Samples were then stained with 1:200 Alexa-Fluor-conjugated secondary antibodies (Life Technologies) at RT for one hour. Nuclei were stained with DAPI. Cells were imaged using the Zeiss LSM980 or LSM880 confocal microscope. Images were processed as described above in PLA section.

### Statistical analyses

For comparisons of two groups, unpaired *t*-tests were applied. For comparisons of more than three groups, One-way ANOVA with the ordinary ANOVA test. These analyses provide *P*-values for each comparison.

## Supporting information

S1 Fig Rab9a knockdown inhibits HPV infection in both HeLa and HaCaT cells. (**A**) HeLa S3 cells were transfected with siNC or two different siRNAs targeting Rab9a (siRab9a and siRab9a-2) and were subjected to Western blot analysis using antibodies recognizing Rab9a and actin as a loading control. (**B**) siRNA-treated cells as described in (**A**) were mock-infected or infected at the MOI of ~2 with HPV16 PsV L2-3XFLAG containing the GFP reporter plasmid. At 48 hpi, GFP fluorescence was determined by flow cytometry. The results are shown as percent relative infectivity (based on mean fluorescence intensity) normalized to siNC treated cells (*right*). Each dot shows the result of an individual experiment. Bars and error bars show mean and standard deviation, respectively. ****, *P* < 0.0001. (**C**) As in (**A**) except using HaCaT cells. (**D**) As in (**B**) except using HaCaT cells.
(TIF)

S2 Fig Rab9a knockdown impairs HPV trafficking from endosomes to Golgi. (**A**) HeLa S3 cells were transfected with siNC or siRab9a siRNAs and infected with HPV harboring the HcRed reporter plasmid at the MOI of ~200. At 16 hpi, PLA was performed with antibodies recognizing FLAG (i.e., HPV L2) and EEA1. Mock, uninfected; HPV, infected. PLA signals are green; nuclei are blue (DAPI). Similar results were obtained in two independent experiments. (**B**) The fluorescence of PLA signals was determined from multiple images obtained as in (**A**). Each dot represents an individual cell (*n*>40) and black horizontal lines indicate the mean value of the analyzed population in each group. ****, *P* < 0.0001; ns, not significant. The graph shows results of a representative experiment. Similar results were obtained in two independent experiments. (**C**) As in (**A**) except PLA was performed with antibodies recognizing FLAG and TGN46. (**D**) Images as in (**C**) were analyzed as described in (**B**). (**E**) As in (**A**) except PLA was performed at 24 hpi with antibodies recognizing FLAG and LAMP1. (**F**) Images as in (**E**) were analyzed as described in (**B**).
(TIF)

S3 Fig HPV L1-Rab9a association requires endosome acidification and γ-secretase activity. (**A**) HeLa S3 cells were infected at the MOI of ~200 with HPV16 PsV L2-3XFLAG containing the HcRed reporter plasmid. DMSO, Bafilomycin A1 (BafA), or γ-secretase inhibitor (XXI) were added to the medium 30 min prior to infection. At 6.5 hpi, PLA was performed with antibodies recognizing HPV L1 and Rab9a. Mock, uninfected. PLA signals are green; nuclei are blue (DAPI). Similar results were obtained in two independent experiments. (**B**) The fluorescence of PLA signals was determined from multiple images obtained as in (**A**). Each dot represents an individual cell (*n*>40) and black horizontal lines indicate the mean value of the analyzed population in each group. ****, *P* < 0.0001. The graph shows results of a representative experiment. Similar results were obtained in two independent experiments.
(TIF)

S4 Fig Knockdown of Rab9a or VPS35 causes HPV accumulation in endosomes. (**A**) HeLa S3 cells were transfected with siNC, siRab9a or siVPS35 and mock-infected or infected at the MOI of ~200 with HPV16 PsV L2-3XFLAG containing the HcRed reporter plasmid. At 16 hpi, PLA was performed with antibodies recognizing HPV L1 and EEA1. Mock, uninfected; HPV, infected. PLA signals are green; nuclei are blue (DAPI). Similar results were obtained in two independent experiments. (**B**) The fluorescence of PLA signals was determined from multiple images obtained as in (**A**). Each dot represents an individual cell (*n*>40) and black horizontal lines indicate the mean value of the analyzed population in each group. ****, *P* < 0.0001. The graph shows results of a representative experiment. Similar results were obtained in two independent experiments.
(TIF)

S5 Fig Rab9a knockdown increases HPV-retromer association. (**A**) HeLa S3 cells were transfected with siNC or siRab9a and infected at the MOI of ~200 with HPV16 PsV L2-3XFLAG containing the HcRed reporter plasmid. At 8 or 16 hpi, PLA was performed with antibodies recognizing HPV L1 and VPS35. Mock, uninfected. PLA signals are green; nuclei are blue (DAPI). Similar results were obtained in two independent experiments. (**B**) The fluorescence of PLA signals was determined from multiple images obtained as in (**A**). Each dot represents an individual cell (n>40) and black horizontal lines indicate the mean value of the analyzed population in each group. NC, siNC; R9, siRab9a. ****, *P* < 0.0001; ns, not significant. The graph shows results of a representative experiment. Similar results were obtained in two independent experiments. (**C**) As in (**A**) at 8 hpi except DMSO, Bafilomycin A1 (BafA), or γ-secretase inhibitor (XXI) were added to the medium 30 min prior to infection. (**D**) Images as in (**C**) were analyzed as described in (**B**). The graph shows results of a representative experiment. Similar results were obtained in two independent experiments.
(TIF)

S6 Fig Rab9a knockdown increases Rab7 association to HPV. (**A**) HeLa S3 cells were transfected with negative control siNC, siRab7, or siRab9a and mock-infected or infected at the MOI of ~200 with HPV16 PsV L2-3XFLAG containing the HcRed reporter plasmid. At 8 hpi, PLA was performed with antibodies recognizing HPV L1 and Rab7. Mock, uninfected; HPV, infected. PLA signals are green; nuclei are blue (DAPI). Similar results were obtained in two independent experiments. (**B**) The fluorescence of PLA signals was determined from multiple images obtained as in (**A**). Each dot represents an individual cell (*n*>25) and black horizontal lines indicate the mean value of the analyzed population in each group. ****, *P* < 0.0001. The graph shows results of a representative experiment. Similar results were obtained in two independent experiments.
(TIF)

S7 Fig Rab9a knockdown increases HPV-retromer association even in cells overexpressing GDP-Rab7. (**A**) HeLa S3 cells stably transduced with an empty vector or a plasmid expressing dominant negative Rab7 (Rab7 DN) were transfected with siNC or siRab9a, and mock-infected or infected at the MOI of ~200 with HPV16 PsV L2-3XFLAG containing the HcRed reporter plasmid. At 8 or 16 hpi, PLA was performed with antibodies recognizing HPV L1 and VPS35. PLA signals are green; nuclei are blue (DAPI). Similar results were obtained in two independent experiments. (**B**) The fluorescence of PLA signals was determined from multiple images obtained as in (**A**). Each dot represents an individual cell (*n*>30) and black horizontal lines indicate the mean value of the analyzed population in each group. ***, *P* < 0.001; ****, *P* < 0.0001. The graph shows results of a representative experiment. Similar results were obtained in two independent experiments.
(TIF)

S8 Fig Expressing DN Rab7 depletes most GTP-Rab7. Lysates from HeLa S3 cells or those stably transduced with an empty vector or a plasmid expressing dominant negative Rab7 (Rab7 DN) were pulled down with GST or GST-RILP (IP) and subjected to Western blot analysis using antibodies recognizing Rab7 or GST together with samples not pulled down (input). Numbers below the bottom panel indicate relative percent abundance of GTP-Rab7 normalized to cells transfected with empty vector (set at 100%).
(TIF)

S9 Fig DENND2D knockdown increases HPV infection in both HeLa and HaCaT cells. (**A**) HeLa S3 cells were transfected with siNC or two different siRNAs targeting DENND2D (siDENND2D and siDENND2D-2) and mock-infected or infected at the MOI of ~0.2 with HPV16 PsV L2-3XFLAG containing the GFP reporter plasmid. At 48 hpi, GFP fluorescence was determined by flow cytometry. The results are shown as percent relative infectivity (based on mean fluorescence intensity) normalized to the siNC treated cells (set at 100%). Each dot shows the result of an individual experiment. Bars and error bars show mean and standard deviation, respectively. *, *P* < 0.05; **, *P* < 0.01. (**B**) As in (**A**) except using HaCaT cells. ***, *P* < 0.001. (**C**) As in (**A**) except cells were infected with HPV18 and HPV5.
(TIF)

S10 Fig GTP-Rab9a promotes trafficking of DMT1-II in HPV infected cells. (**A**) 293TT cells were transfected with a plasmid expressing HA-Rab9a CA, followed by transfection of a DMT1-II expressing plasmid 24 h after the first transfection. Where indicated, cells were infected at the MOI of ~100 with HPV16 PsV L2-3XFLAG containing the HcRed reporter plasmid at 8 h after the second transfection. Expression of HA-Rab9a CA was determined by anti-HA staining, which distinguishes cells expressing HA-Rab9a CA (+) or those not expressing it (-). DMT1-II and TGN46 were stained using antibodies recognizing GFP and TGN46, respectively. Immunofluorescence (IF) images were shown; HA-Rab9a CA, orange; TGN46, magenta; DMT1-II, green; nuclei (DAPI), blue. Merged image shows TGN46 and DMT1-II with overlap colored white. Similar results were obtained in two independent experiments. (**B**) Pearson’s correlation coefficient values for TGN46 and DMT1-II colocalization in those cells are shown. Each dot represents an individual cell (n>25) and black horizontal lines indicate the mean value of the analyzed population in each group. ****, *P* < 0.0001. The graph shows results of a representative experiment. Similar results were obtained in two independent experiments.
(TIF)

S1 Table Oligonucleotides used in this study. (XLSX)
