# Supplementary figures and images for: Noncanonical Rab9a action supports retromer-mediated endosomal exit of human papillomavirus during virus entry
Source: PLoS Pathog. 2023 Sep 13;19(9):e1011648. doi: 10.1371/journal.ppat.1011648 (PMC10519607; doi:10.1371/journal.ppat.1011648)

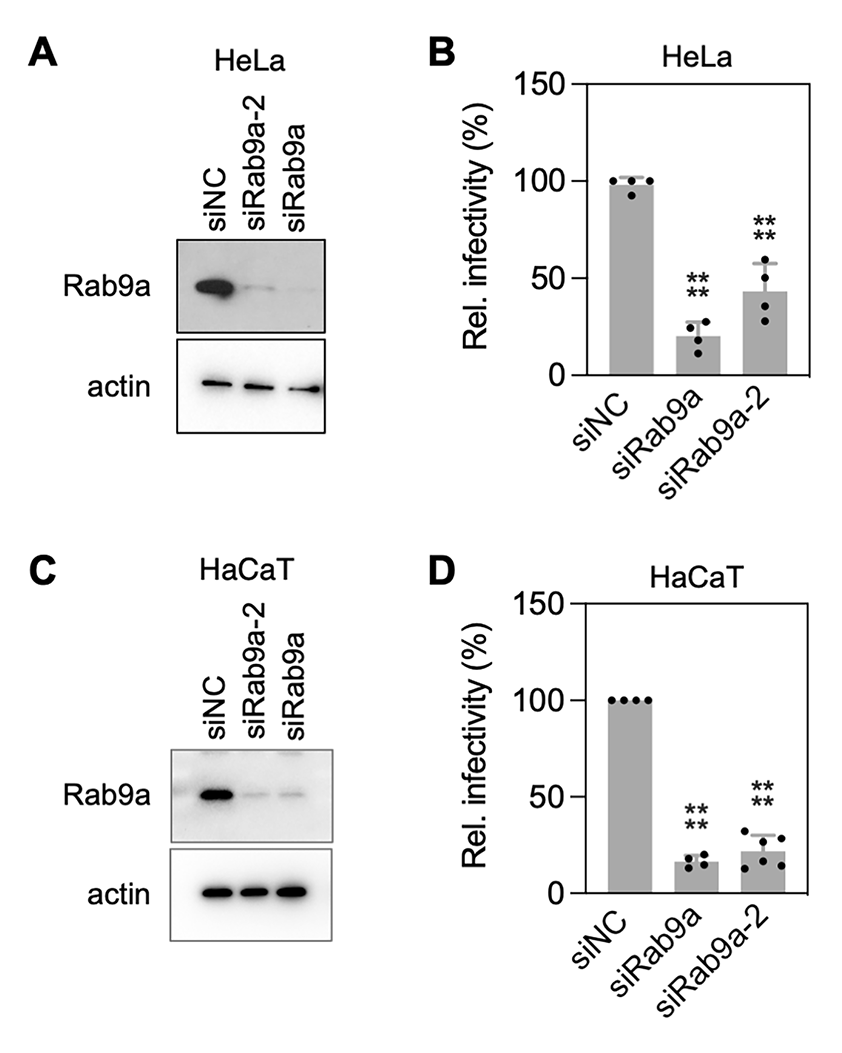

Supplement: S1 Fig — (A) HeLa S3 cells were transfected with siNC or two different siRNAs targeting Rab9a (siRab9a and siRab9a-2) and were subjected to Western blot analysis using antibodies recognizing Rab9a and actin as a loading control. (B) siRNA-treated cells as described in (A) were mock-infected or infected at the MOI of ~2 with HPV16 PsV L2-3XFLAG containing the GFP reporter plasmid. At 48 hpi, GFP fluorescence was determined by flow cytometry. The results are shown as percent relative infectivity (based on mean fluorescence intensity) normalized to siNC treated cells (right). Each dot shows the result of an individual experiment. Bars and error bars show mean and standard deviation, respectively. ****, P < 0.0001. (C) As in (A) except using HaCaT cells. (D) As in (B) except using HaCaT cells. (TIF) [file ppat.1011648.s001.tif]

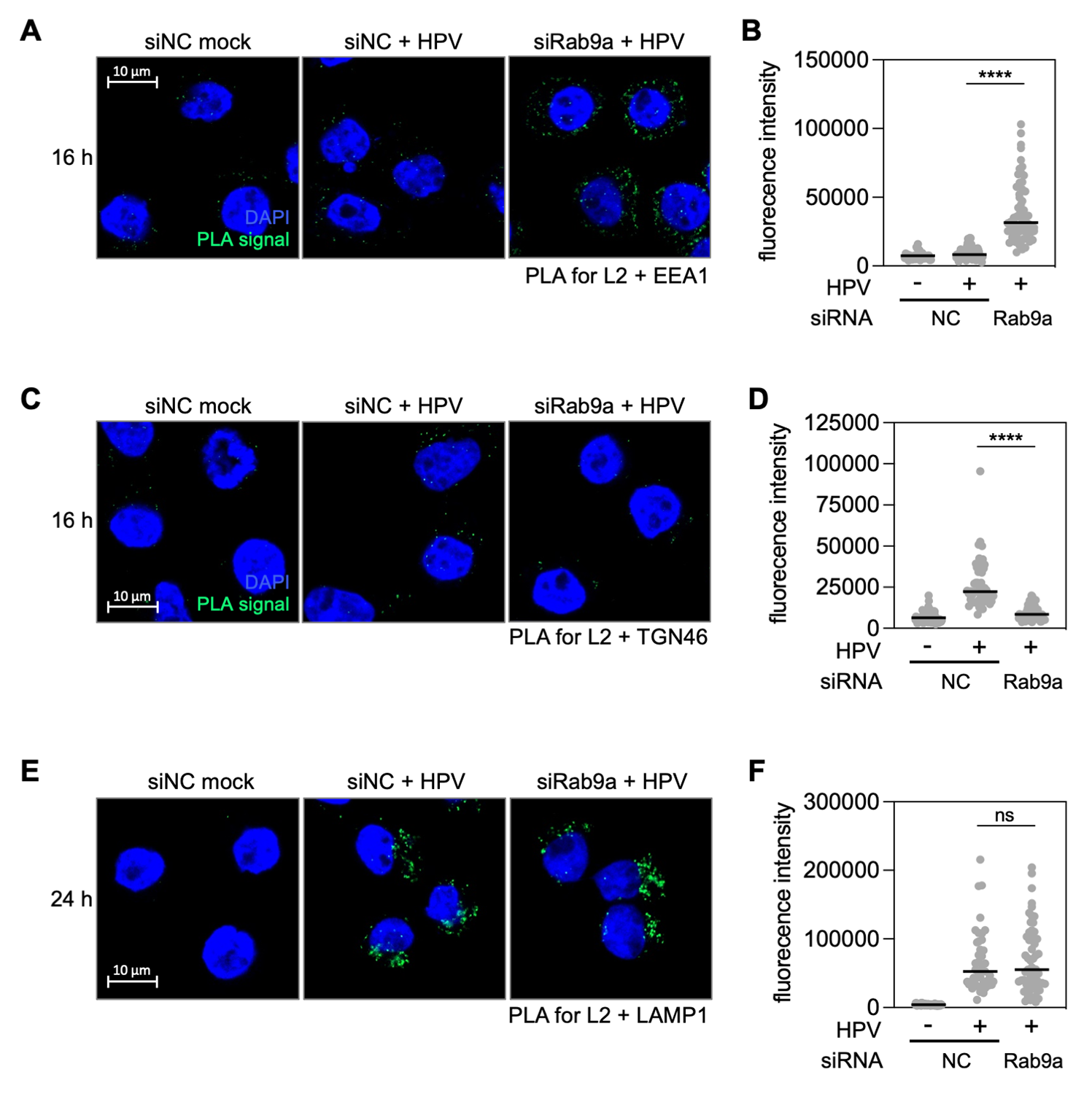

Supplement: S2 Fig — (A) HeLa S3 cells were transfected with siNC or siRab9a siRNAs and infected with HPV harboring the HcRed reporter plasmid at the MOI of ~200. At 16 hpi, PLA was performed with antibodies recognizing FLAG (i.e., HPV L2) and EEA1. Mock, uninfected; HPV, infected. PLA signals are green; nuclei are blue (DAPI). Similar results were obtained in two independent experiments. (B) The fluorescence of PLA signals was determined from multiple images obtained as in (A). Each dot represents an individual cell (n>40) and black horizontal lines indicate the mean value of the analyzed population in each group. ****, P < 0.0001; ns, not significant. The graph shows results of a representative experiment. Similar results were obtained in two independent experiments. (C) As in (A) except PLA was performed with antibodies recognizing FLAG and TGN46. (D) Images as in (C) were analyzed as described in (B). (E) As in (A) except PLA was performed at 24 hpi with antibodies recognizing FLAG and LAMP1. (F) Images as in (E) were analyzed as described in (B). (TIF) [file ppat.1011648.s002.tif]

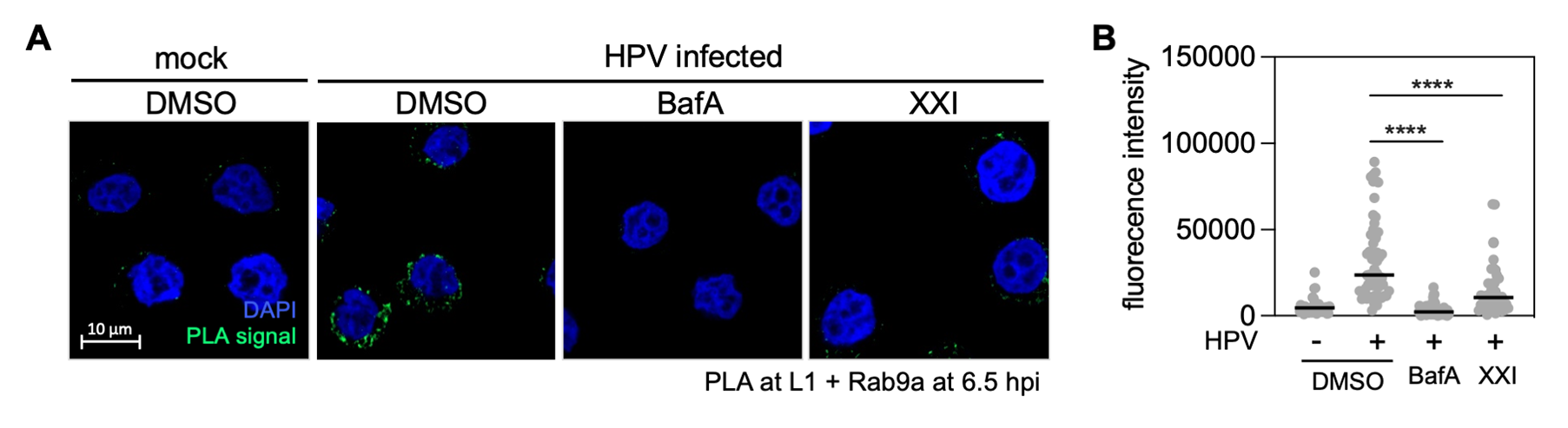

Supplement: S3 Fig — (A) HeLa S3 cells were infected at the MOI of ~200 with HPV16 PsV L2-3XFLAG containing the HcRed reporter plasmid. DMSO, Bafilomycin A1 (BafA), or γ-secretase inhibitor (XXI) were added to the medium 30 min prior to infection. At 6.5 hpi, PLA was performed with antibodies recognizing HPV L1 and Rab9a. Mock, uninfected. PLA signals are green; nuclei are blue (DAPI). Similar results were obtained in two independent experiments. (B) The fluorescence of PLA signals was determined from multiple images obtained as in (A). Each dot represents an individual cell (n>40) and black horizontal lines indicate the mean value of the analyzed population in each group. ****, P < 0.0001. The graph shows results of a representative experiment. Similar results were obtained in two independent experiments. (TIF) [file ppat.1011648.s003.tif]

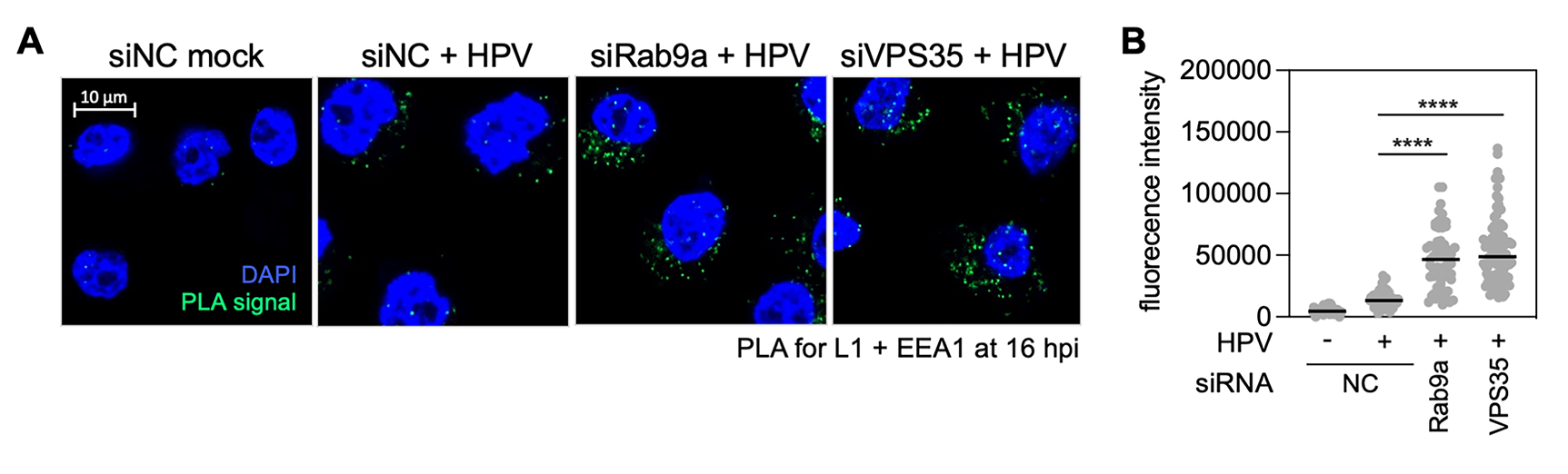

Supplement: S4 Fig — (A) HeLa S3 cells were transfected with siNC, siRab9a or siVPS35 and mock-infected or infected at the MOI of ~200 with HPV16 PsV L2-3XFLAG containing the HcRed reporter plasmid. At 16 hpi, PLA was performed with antibodies recognizing HPV L1 and EEA1. Mock, uninfected; HPV, infected. PLA signals are green; nuclei are blue (DAPI). Similar results were obtained in two independent experiments. (B) The fluorescence of PLA signals was determined from multiple images obtained as in (A). Each dot represents an individual cell (n>40) and black horizontal lines indicate the mean value of the analyzed population in each group. ****, P < 0.0001. The graph shows results of a representative experiment. Similar results were obtained in two independent experiments. (TIF) [file ppat.1011648.s004.tif]

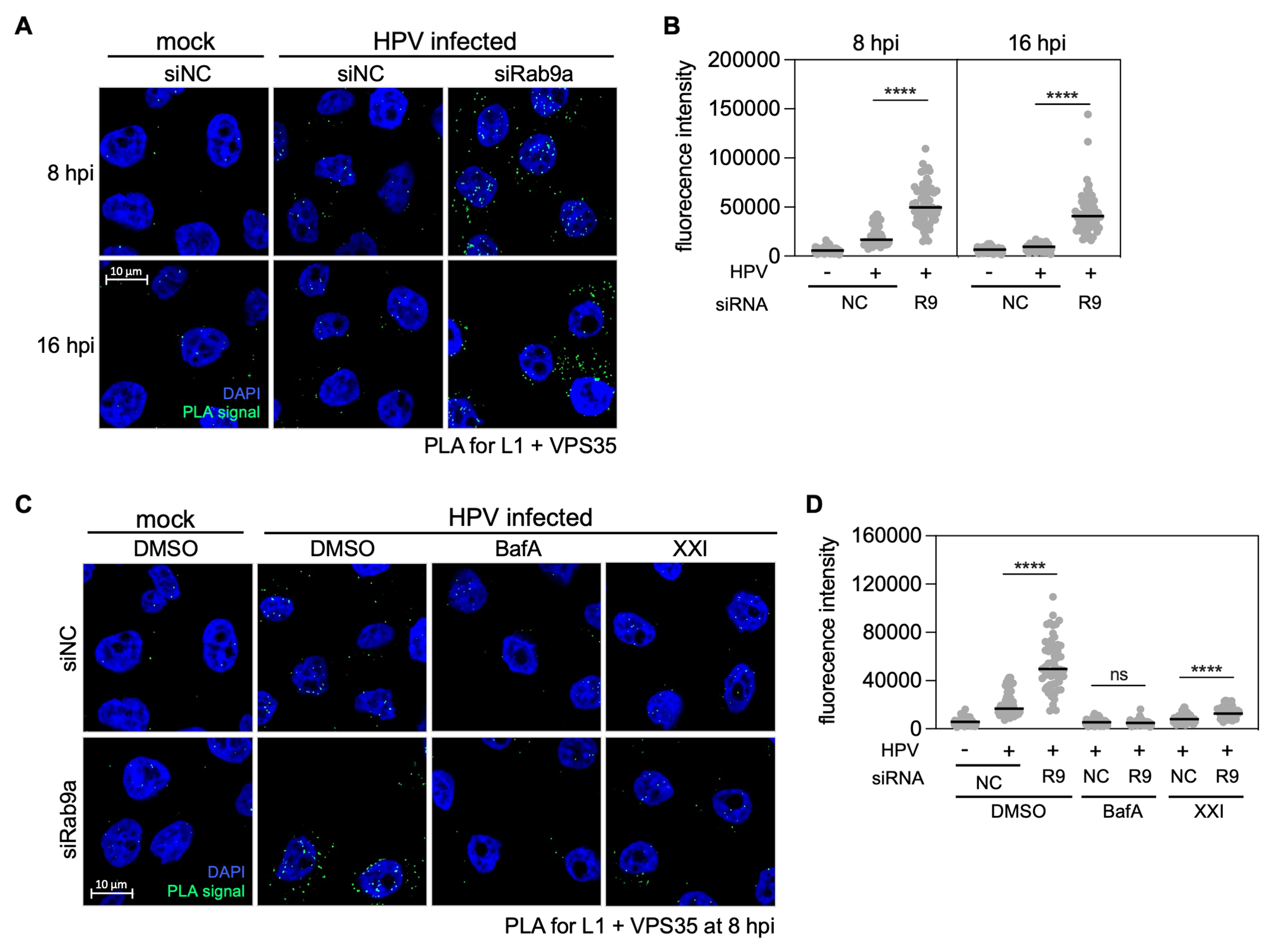

Supplement: S5 Fig — (A) HeLa S3 cells were transfected with siNC or siRab9a and infected at the MOI of ~200 with HPV16 PsV L2-3XFLAG containing the HcRed reporter plasmid. At 8 or 16 hpi, PLA was performed with antibodies recognizing HPV L1 and VPS35. Mock, uninfected. PLA signals are green; nuclei are blue (DAPI). Similar results were obtained in two independent experiments. (B) The fluorescence of PLA signals was determined from multiple images obtained as in (A). Each dot represents an individual cell (n>40) and black horizontal lines indicate the mean value of the analyzed population in each group. NC, siNC; R9, siRab9a. ****, P < 0.0001; ns, not significant. The graph shows results of a representative experiment. Similar results were obtained in two independent experiments. (C) As in (A) at 8 hpi except DMSO, Bafilomycin A1 (BafA), or γ-secretase inhibitor (XXI) were added to the medium 30 min prior to infection. (D) Images as in (C) were analyzed as described in (B). The graph shows results of a representative experiment. Similar results were obtained in two independent experiments. (TIF) [file ppat.1011648.s005.tif]

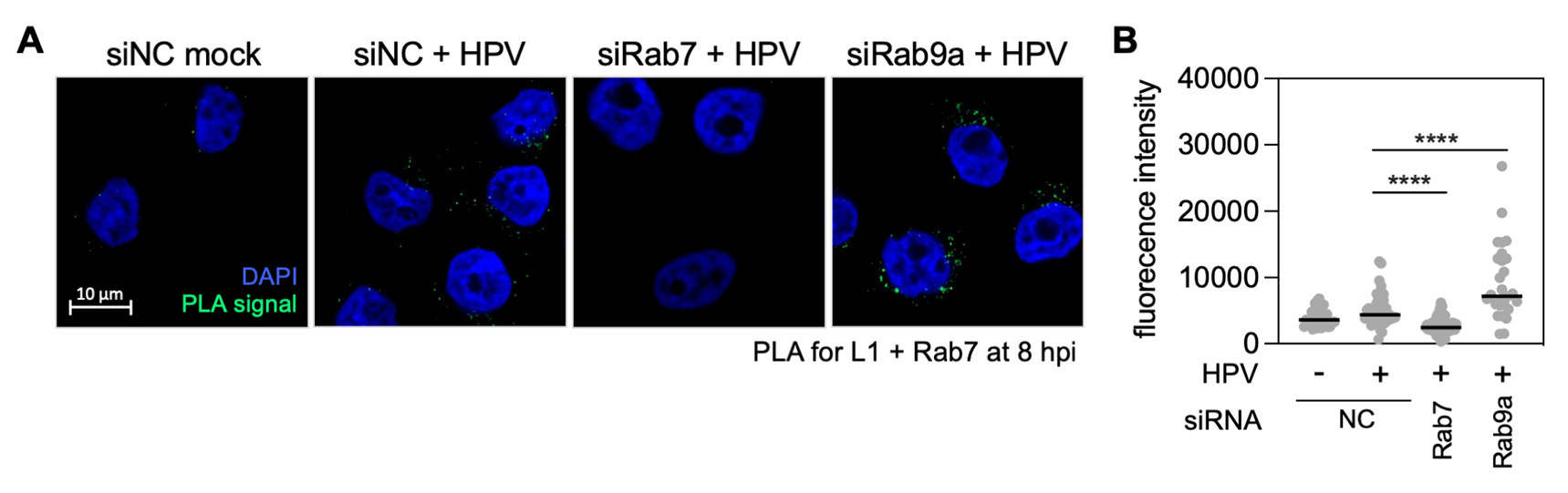

Supplement: S6 Fig — (A) HeLa S3 cells were transfected with negative control siNC, siRab7, or siRab9a and mock-infected or infected at the MOI of ~200 with HPV16 PsV L2-3XFLAG containing the HcRed reporter plasmid. At 8 hpi, PLA was performed with antibodies recognizing HPV L1 and Rab7. Mock, uninfected; HPV, infected. PLA signals are green; nuclei are blue (DAPI). Similar results were obtained in two independent experiments. (B) The fluorescence of PLA signals was determined from multiple images obtained as in (A). Each dot represents an individual cell (n>25) and black horizontal lines indicate the mean value of the analyzed population in each group. ****, P < 0.0001. The graph shows results of a representative experiment. Similar results were obtained in two independent experiments. (TIF) [file ppat.1011648.s006.tif]

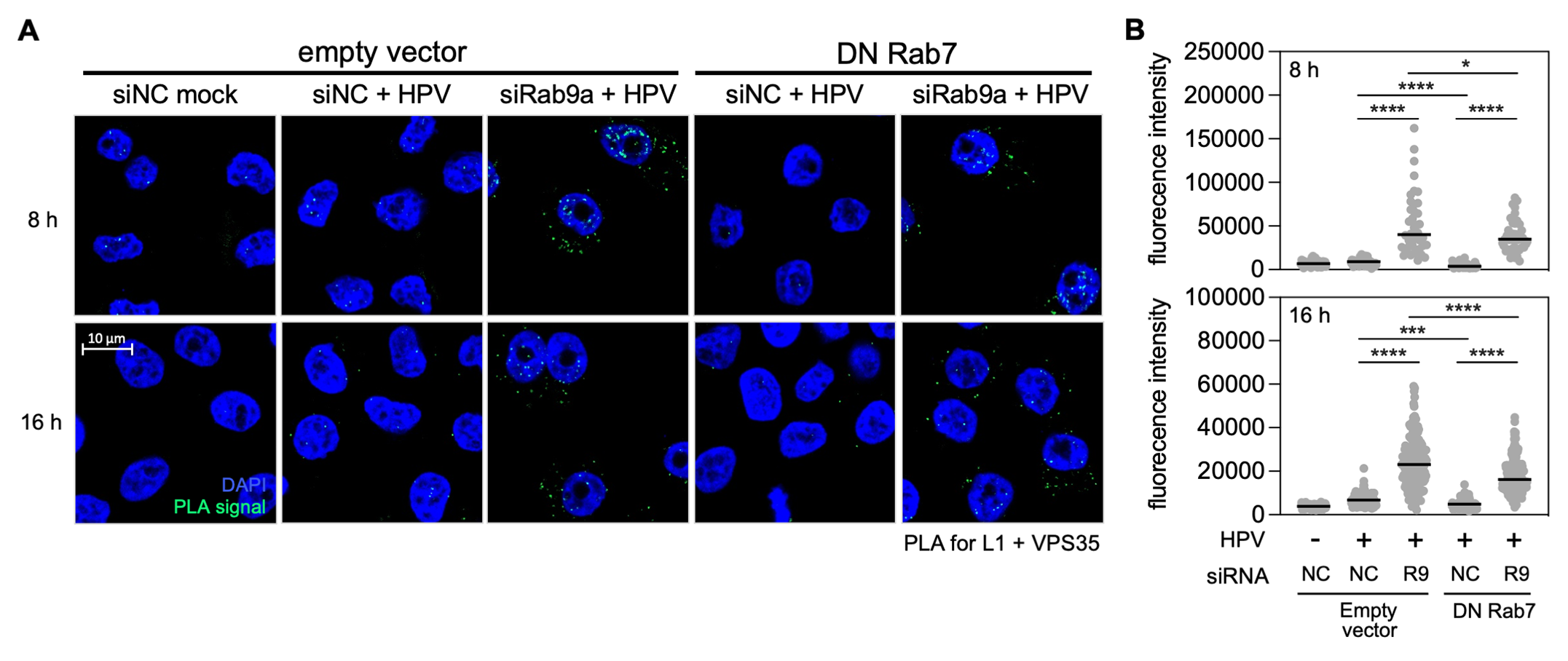

Supplement: S7 Fig — (A) HeLa S3 cells stably transduced with an empty vector or a plasmid expressing dominant negative Rab7 (Rab7 DN) were transfected with siNC or siRab9a, and mock-infected or infected at the MOI of ~200 with HPV16 PsV L2-3XFLAG containing the HcRed reporter plasmid. At 8 or 16 hpi, PLA was performed with antibodies recognizing HPV L1 and VPS35. PLA signals are green; nuclei are blue (DAPI). Similar results were obtained in two independent experiments. (B) The fluorescence of PLA signals was determined from multiple images obtained as in (A). Each dot represents an individual cell (n>30) and black horizontal lines indicate the mean value of the analyzed population in each group. ***, P < 0.001; ****, P < 0.0001. The graph shows results of a representative experiment. Similar results were obtained in two independent experiments. (TIF) [file ppat.1011648.s007.tif]

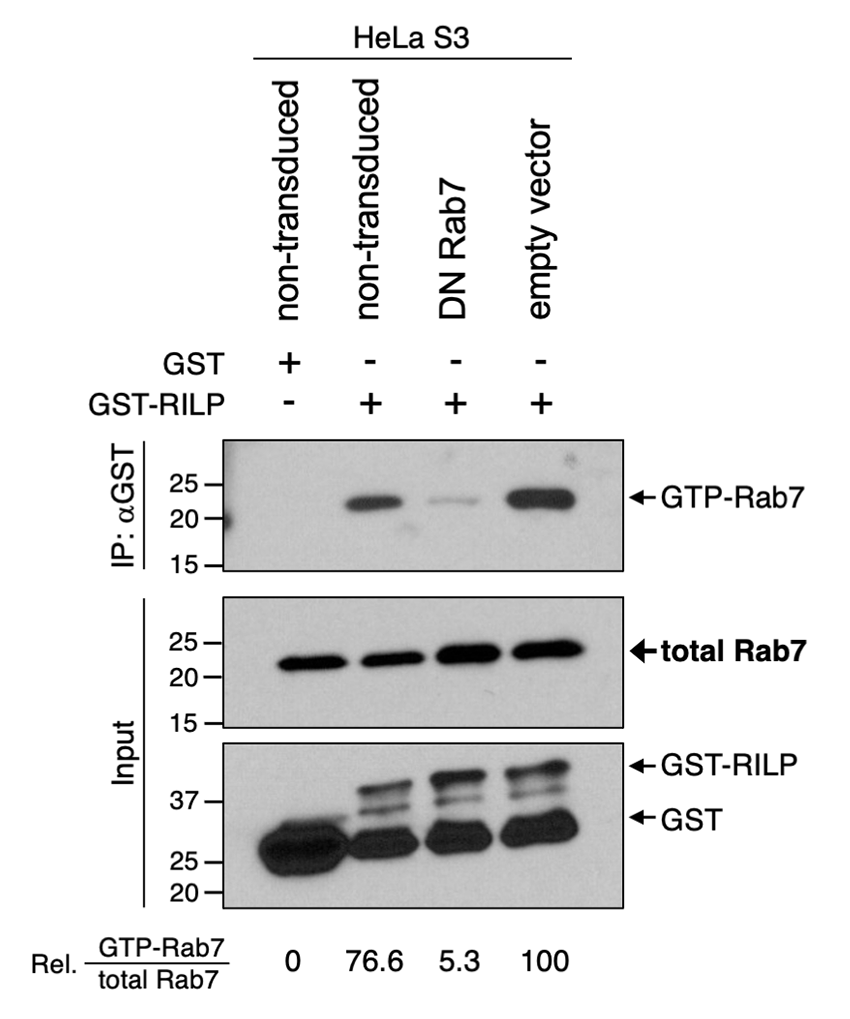

Supplement: S8 Fig — Lysates from HeLa S3 cells or those stably transduced with an empty vector or a plasmid expressing dominant negative Rab7 (Rab7 DN) were pulled down with GST or GST-RILP (IP) and subjected to Western blot analysis using antibodies recognizing Rab7 or GST together with samples not pulled down (input). Numbers below the bottom panel indicate relative percent abundance of GTP-Rab7 normalized to cells transfected with empty vector (set at 100%). (TIF) [file ppat.1011648.s008.tif]

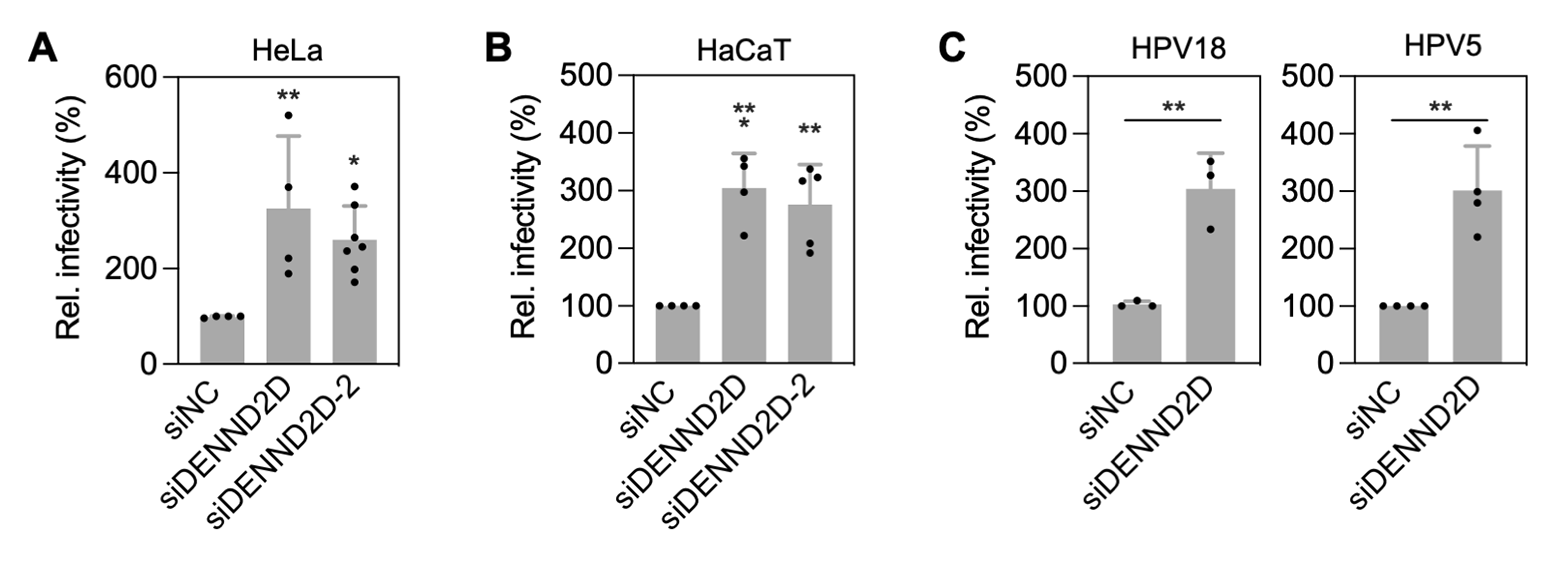

Supplement: S9 Fig — (A) HeLa S3 cells were transfected with siNC or two different siRNAs targeting DENND2D (siDENND2D and siDENND2D-2) and mock-infected or infected at the MOI of ~0.2 with HPV16 PsV L2-3XFLAG containing the GFP reporter plasmid. At 48 hpi, GFP fluorescence was determined by flow cytometry. The results are shown as percent relative infectivity (based on mean fluorescence intensity) normalized to the siNC treated cells (set at 100%). Each dot shows the result of an individual experiment. Bars and error bars show mean and standard deviation, respectively. *, P < 0.05; **, P < 0.01. (B) As in (A) except using HaCaT cells. ***, P < 0.001. (C) As in (A) except cells were infected with HPV18 and HPV5. (TIF) [file ppat.1011648.s009.tif]

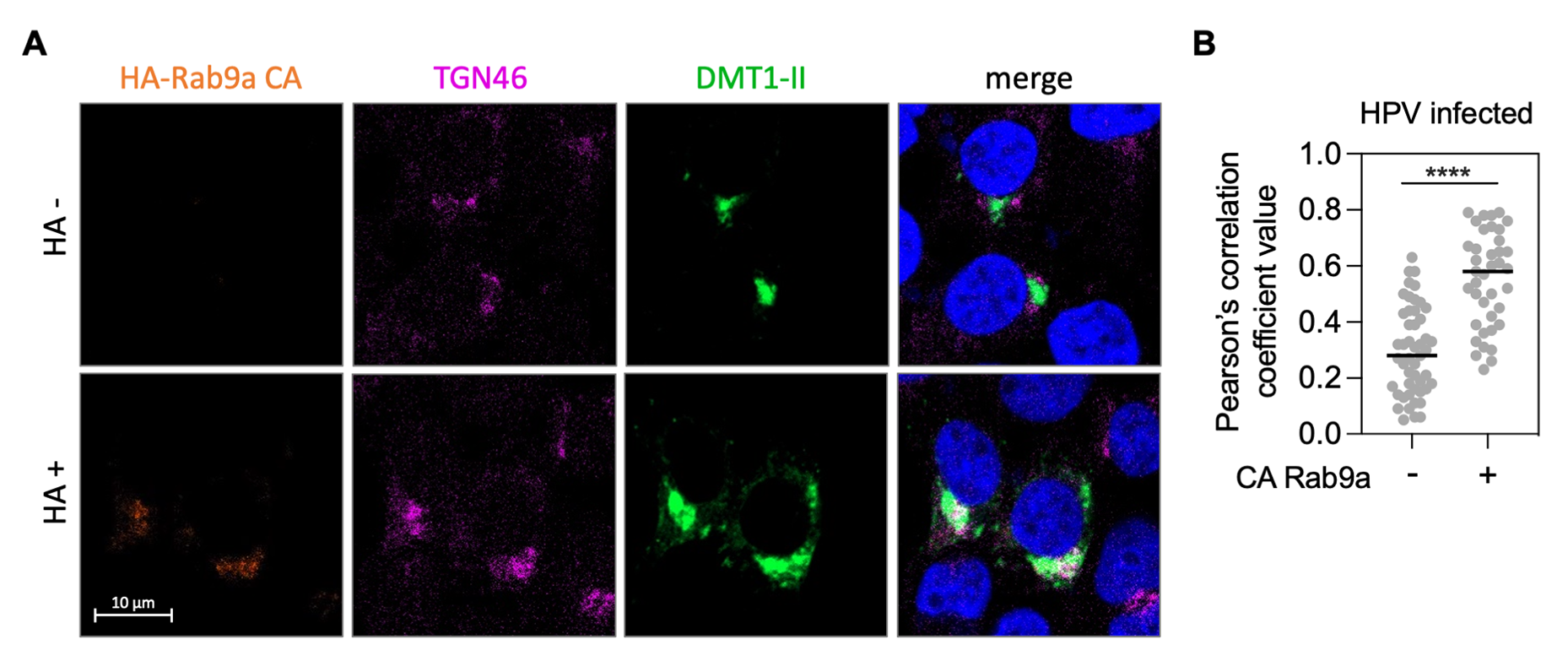

Supplement: S10 Fig — (A) 293TT cells were transfected with a plasmid expressing HA-Rab9a CA, followed by transfection of a DMT1-II expressing plasmid 24 h after the first transfection. Where indicated, cells were infected at the MOI of ~100 with HPV16 PsV L2-3XFLAG containing the HcRed reporter plasmid at 8 h after the second transfection. Expression of HA-Rab9a CA was determined by anti-HA staining, which distinguishes cells expressing HA-Rab9a CA (+) or those not expressing it (-). DMT1-II and TGN46 were stained using antibodies recognizing GFP and TGN46, respectively. Immunofluorescence (IF) images were shown; HA-Rab9a CA, orange; TGN46, magenta; DMT1-II, green; nuclei (DAPI), blue. Merged image shows TGN46 and DMT1-II with overlap colored white. Similar results were obtained in two independent experiments. (B) Pearson’s correlation coefficient values for TGN46 and DMT1-II colocalization in those cells are shown. Each dot represents an individual cell (n>25) and black horizontal lines indicate the mean value of the analyzed population in each group. ****, P < 0.0001. The graph shows results of a representative experiment. Similar results were obtained in two independent experiments. (TIF) [file ppat.1011648.s010.tif]
